# Supplementary material for: A facile one step route that introduces functionality to polymer powders for laser sintering
Source: Nat Commun. 2024 Apr 11;15:3137. doi: 10.1038/s41467-024-47376-4 (PMC11009337; doi:10.1038/s41467-024-47376-4)
Supplement: Supplementary file 1 — Supplementary Information [file 41467_2024_47376_MOESM1_ESM.pdf]

Supplementary Information

A facile one step route that introduces functionality to polymer powders for Laser Sintering

Eduards Krumins[1], Liam A. Crawford[2], David M. Rogers[1], Fabricio Machado[1,3], Vincenzo Taresco[1], Mark East[4], Samuel Irving[1], Harriet R. Fowler[1], Long Jiang[5], Nichola Starr[5], Christopher Parmenter[6], Kristoffer Kortsen[1], Valentina Cuzzucoli Crucitti[4], Simon V. Avery[2], Christopher Tuck[4] & *Steven M. Howdle[1]*

[1]School of Chemistry, University of Nottingham, University Park Nottingham, NG7 2RD, United Kingdom

[2]Life Sciences, Faculty of Medicine and Health Sciences, University of Nottingham, University Park Nottingham, NG7 2RD, United Kingdom

[3] Institute of Chemistry, University of Brasília, Campus Universitário Darcy Ribeiro, 70910-900, Brasília, DF, Brazil

[4]Centre of Additive Manufacturing, Faculty of Engineering, University of Nottingham, 522 Derby Rd, Lenton, Nottingham NG7 2GX, United Kingdom

[5]School of Pharmacy, University of Nottingham, University Park Nottingham, NG7 2RD, United Kingdom

[6]Nottingham Nanoscale and Microscale Research Centre, University Park, University of Nottingham, Nottingham, NG7 2RD, UK, United Kingdom

Table of Contents:

Supplementary Methods……………………………………………………………………3-4

Supplementary Discussion…………………………………………………………………4-55

References……………………………………………………………………………………55

Supplementary Methods:

Synthesis of Disperse Red 1 Methacrylate (DR1MA)

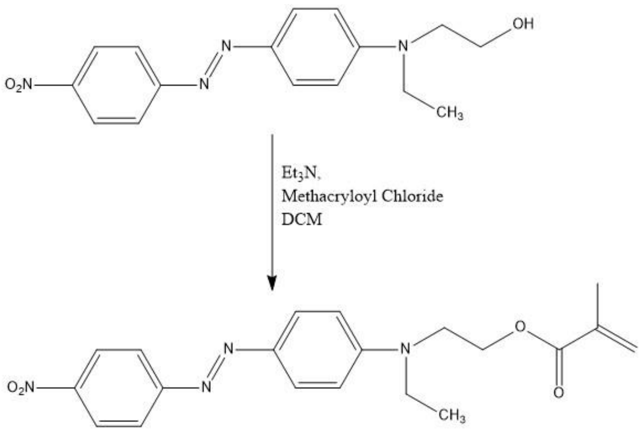

Supplementary Fig 1 – Reaction scheme for the synthesis of Disperse Red 1 Methacrylate

Synthesis of Yellow 1 Methacrylate (Y1MA):

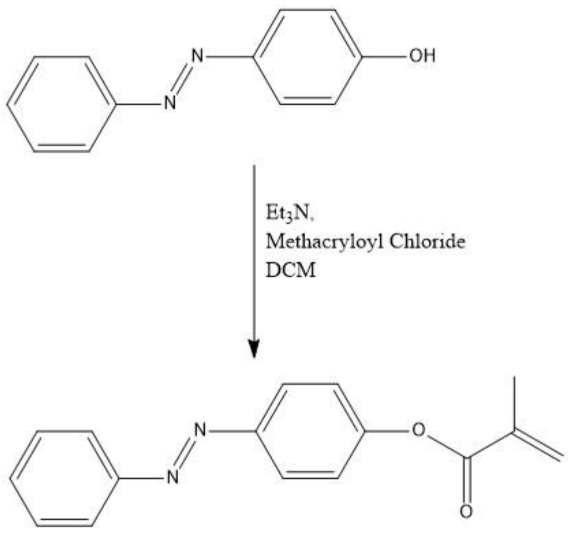

Supplementary Fig 2 - Reaction scheme for the synthesis of Yellow 1 Methacrylate

Synthesis of disperse blue 3 methacrylate (DB3MA):

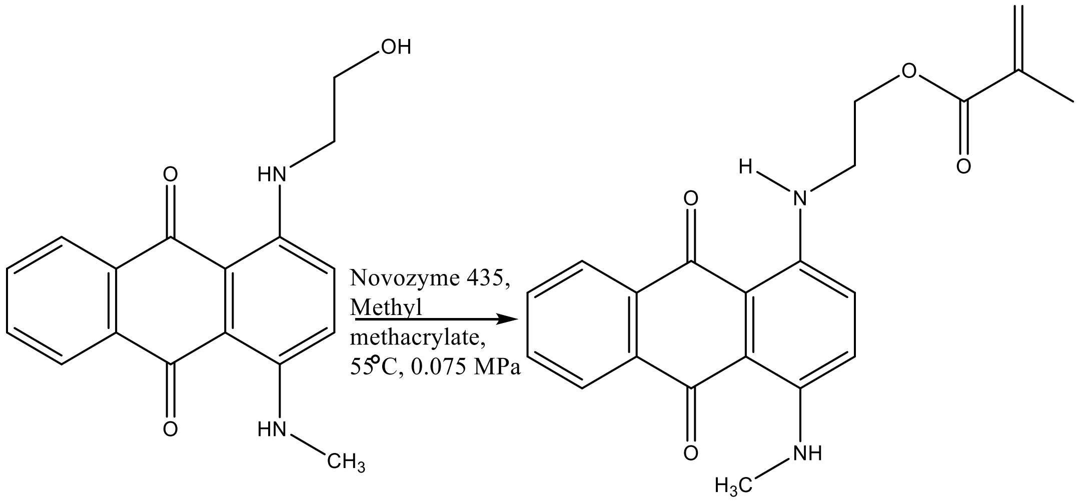

Supplementary Fig 3 - Reaction scheme for the synthesis of Disperse Blue 3 Methacrylate 3

Supplementary Discussion:

Supporting information for dye monomer colour modelling and synthesis:
Density functional theory (DFT) was employed using the B3LYP functional with the 6-311G(d,p) basis set for geometry optimisation and subsequent harmonic vibrational frequency calculation. Time-dependent DFT (TDDFT) calculations employed the CAM-B3LYP functional with the 6-311+G(d,p) basis set to compute vertical electronic transition energies. DFT and TDDFT calculations on the chemical species were performed for the gas-phase (vacuum) and in solvent, where the C-PCM model was used to describe the solvent dichloromethane (DCM). TDDFT calculations employed the Tamm-Dancoff approximation, and the equations were solved for 10 roots (Supplementary Tables 1 and 2). The monomers were then synthesized and analysed via ^{1}H NMR (Supplementary Fig 5-7).

The ORCA program version 4.0.1.2 was employed for all computations.

**Compound**	**ORCA output file**	**Energy (cm^{-1})**	**Wavelength (nm)**	***f* (a.u.)**
**Gas phase**				
Azobenzene hydroxy	azoben_hydroxy_td.out.2123061	23138.7<br>32425.1	432.2<br>308.4	0.0<br>1.1106
Azobenzene methacrylate	azoben_methacrylate_td.out.2590375	22756.8<br>32718.4	439.4<br>305.6	0.0<br>1.2734
**Solvent**				
Anthraquinone hydroxy	anthra_hydroxy_td_dcm.out.4354736	21372.5<br>28569.6	467.9<br>350.0	0.4308<br>0.0001
Anthraquinone methacrylate	anthra_methacr_td_dcm.out.4355078	21398.6<br>28583.7	467.3<br>349.9	0.4340<br>0.0001
Azobenzene hydroxy	azoben_hydroxy_td_dcm.out.2790644	23266.5<br>31109.0	429.8<br>321.5	0.0<br>1.0770
Azobenzene methacrylate	azoben_methacrylate_td_dcm.out.2790666	22826.3<br>31863.6	438.1<br>313.8	0.0<br>1.2468

Supplementary Table 1 - Computed vertical transition energy and oscillator strength (*f*) for the first and second excited states of each of the anthraquinone and azobenzene compounds in gas phase and in solvent (DCM) using the gas-phase optimised geometries.

**Compound**	**ORCA output file**	**Energy (cm^{-1})**	**Wavelength (nm)**	***f* (a.u.)**
**Solvent**				
Azobenzene hydroxy	azoben_hydroxy_td_dcm-op.out.4354079	23069.3<br>31007.5	433.5<br>322.5	0.0<br>1.0948
Azobenzene methacrylate	azoben_methacrylate_td_dcm-op.out.4354082	22661.0<br>32336.6	441.3<br>309.2	0.0001<br>1.2005

Supplementary Table 2 **-** Computed vertical transition energy and oscillator strength (*f*) for the first and second excited states of each of the azobenzene compounds in solvent (DCM) using the solvent optimised geometries.

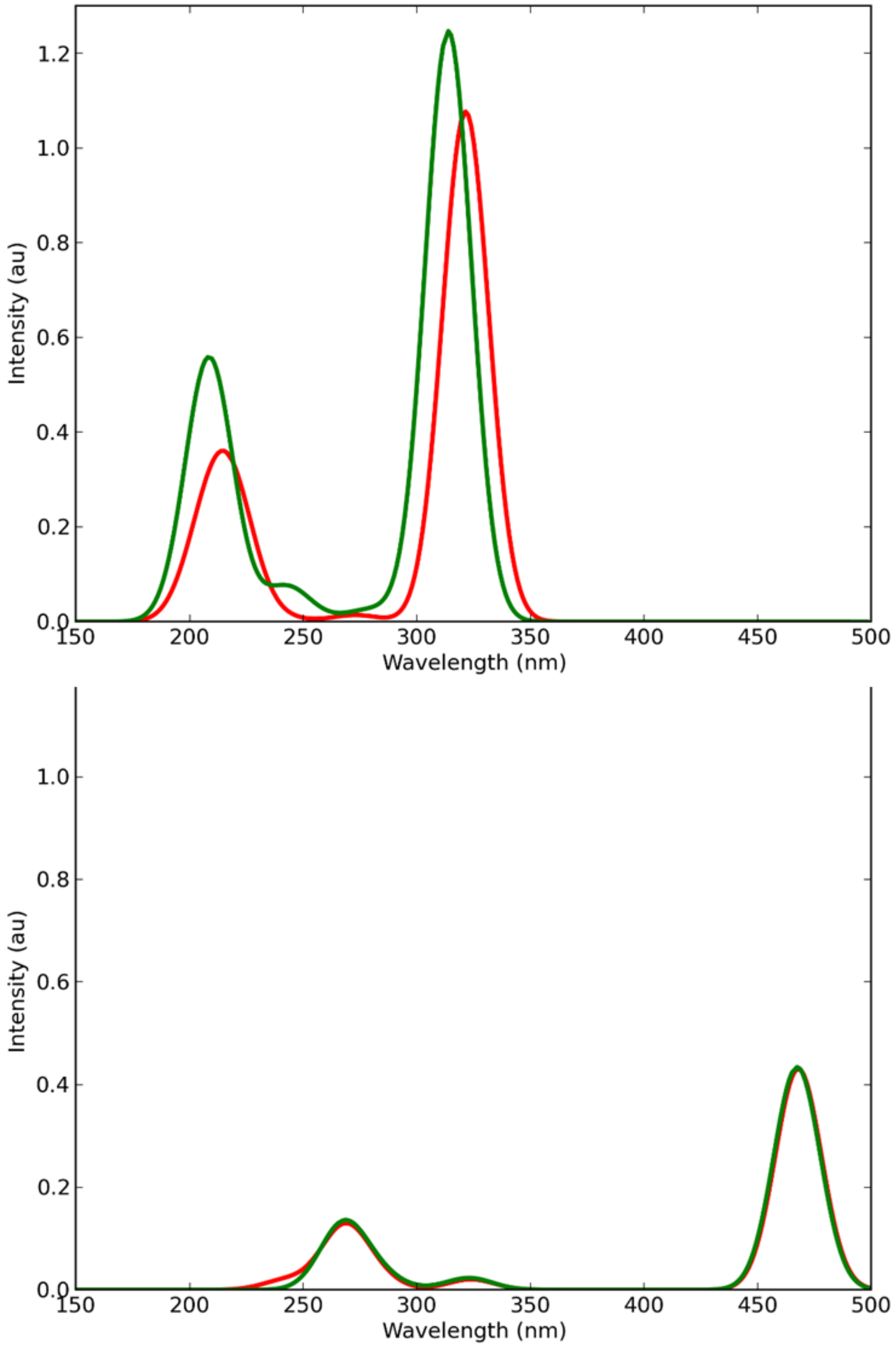

Supplementary Fig 4 **-** Computed UV spectra for azobenzene hydroxy (red line) and azobenzene methacrylate (green line, upper panel), and anthraquinone hydroxy (red line) and anthraquinone methacrylate (green line, lower panel). Gaussian functions of half width 12.0 nm were fitted to the TDDFT calculated vertical transitions for the gas-phase optimised geometries in solvent (DCM).

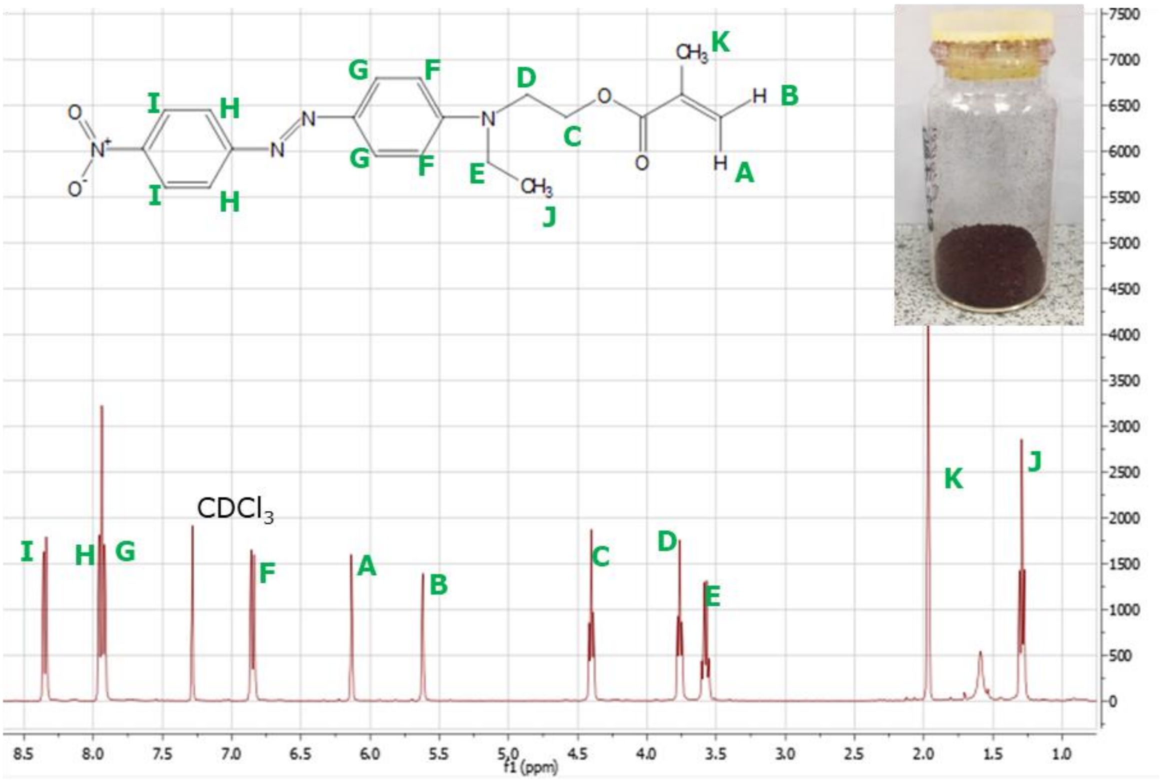

Supplementary Fig 5 – ^{1}H NMR of Disperse Red 1 methacrylate.

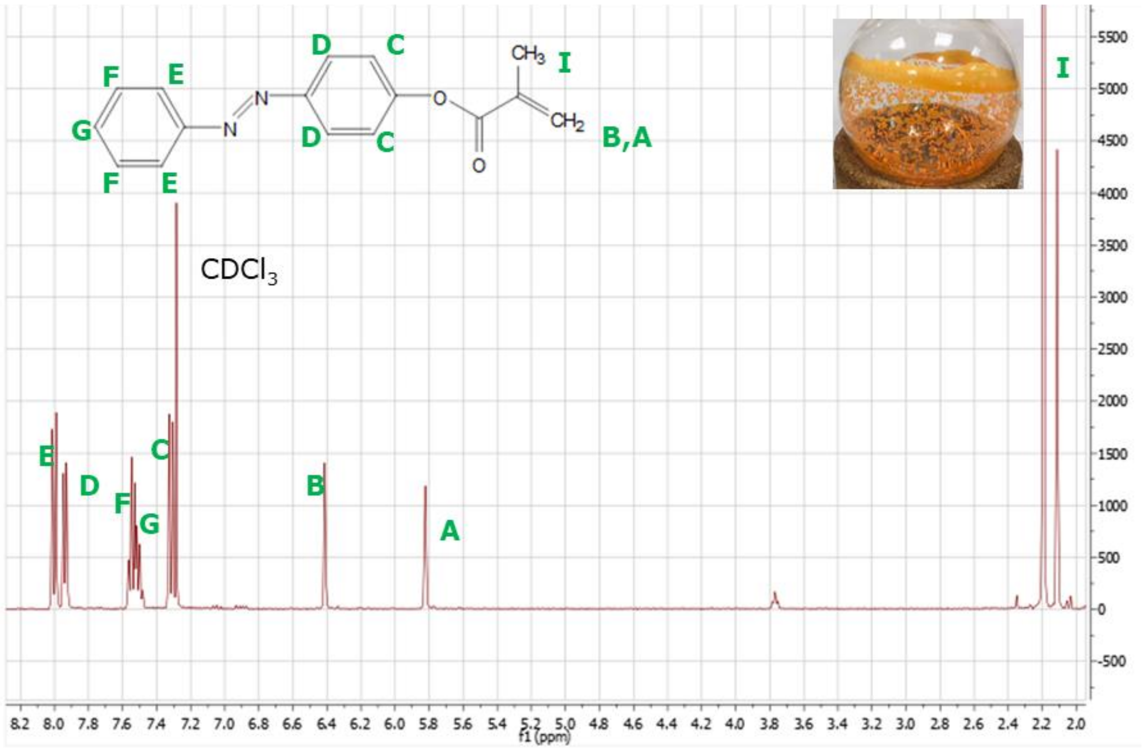

Supplementary Fig 6 - ^{1}H NMR of Yellow 1 methacrylate.

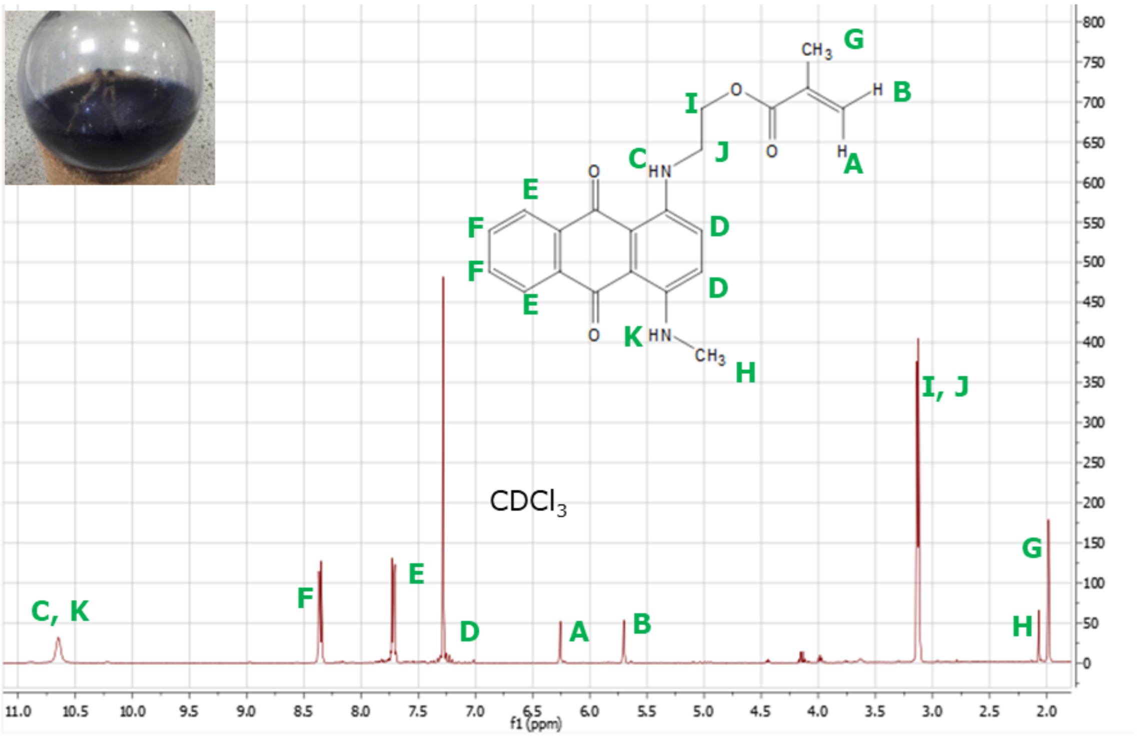

Supplementary Fig 7 - ^{1}H NMR of Disperse Blue 3 methacrylate.

Supporting information for Polymer Synthesis and Scale up:

To produce coloured materials PA-12 based materials for SLS, PA-12 particles were coated with p(IBMA-dye monomer) through a novel and versatile $scCO_2$ based polymerisation and coating system. At first these reactions were tested and optimized in autoclaves which had a reactor volume of 60 ml. GPC, ^{1}H NMR, DSC, and LDS analysis can be seen for PA-12 coated in p(IBMA-DR1MA), p(IBMA-Y1MA), and p(IBMA-DB3MA) (Supplementary Fig 8-23).

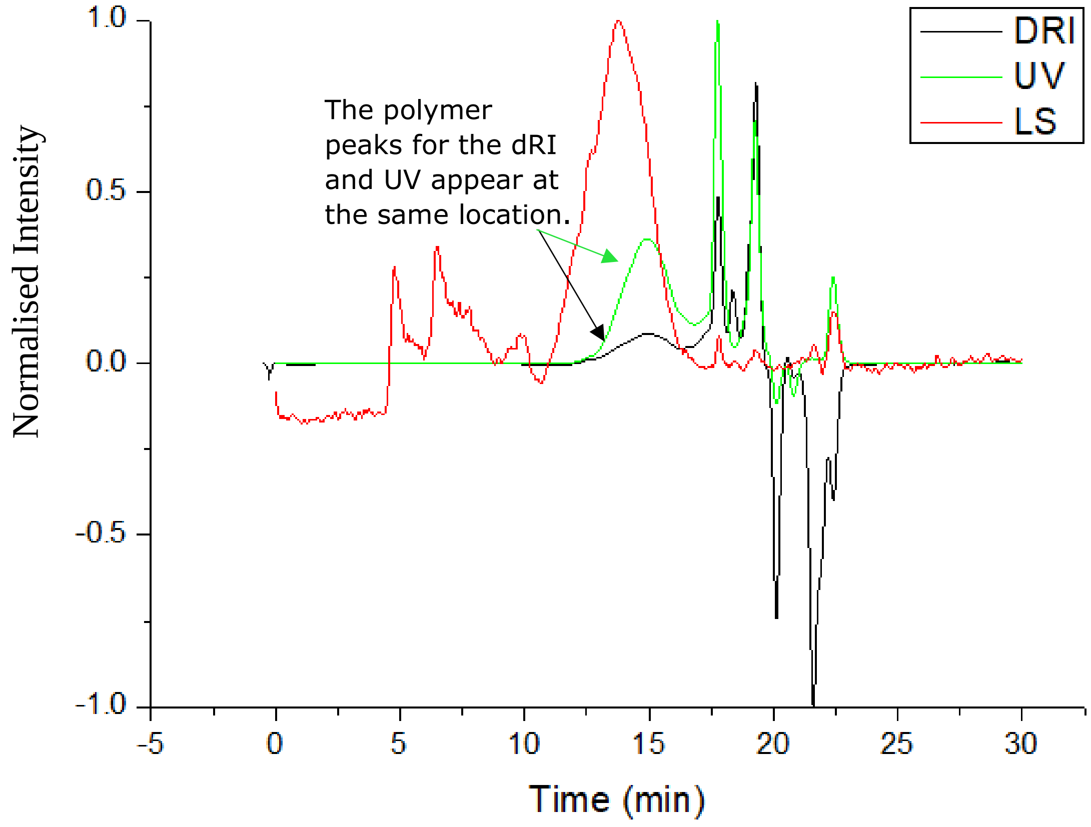

Supplementary Fig 8 – GPC Chromatogram of P(IBMA-Y1MA) outer coating extracted from PA-12 particles coated with P(IBMA-DR1MA) synthesized in a 60 mL autoclave. Showing that UV (ultraviolet) and dRI (differential refractive index) peaks correspond to the same species, demonstrating successful co-polymerisation. LS (light scattering) also has a polymer peak at the same timepoint.

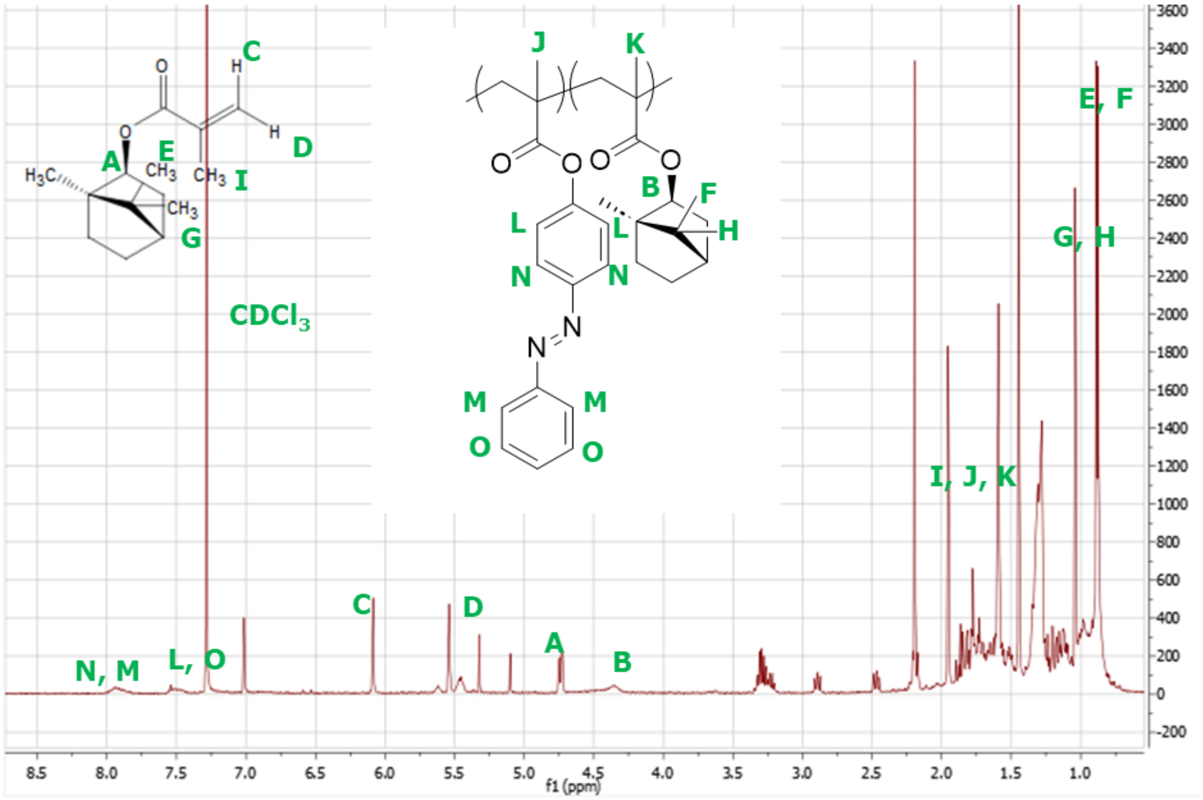

Supplementary Fig 9 – ^{1}H NMR of P(IBMA-Y1MA), showing residues of IBMA and the corresponding polymer peaks for P(IBMA-Y1MA).

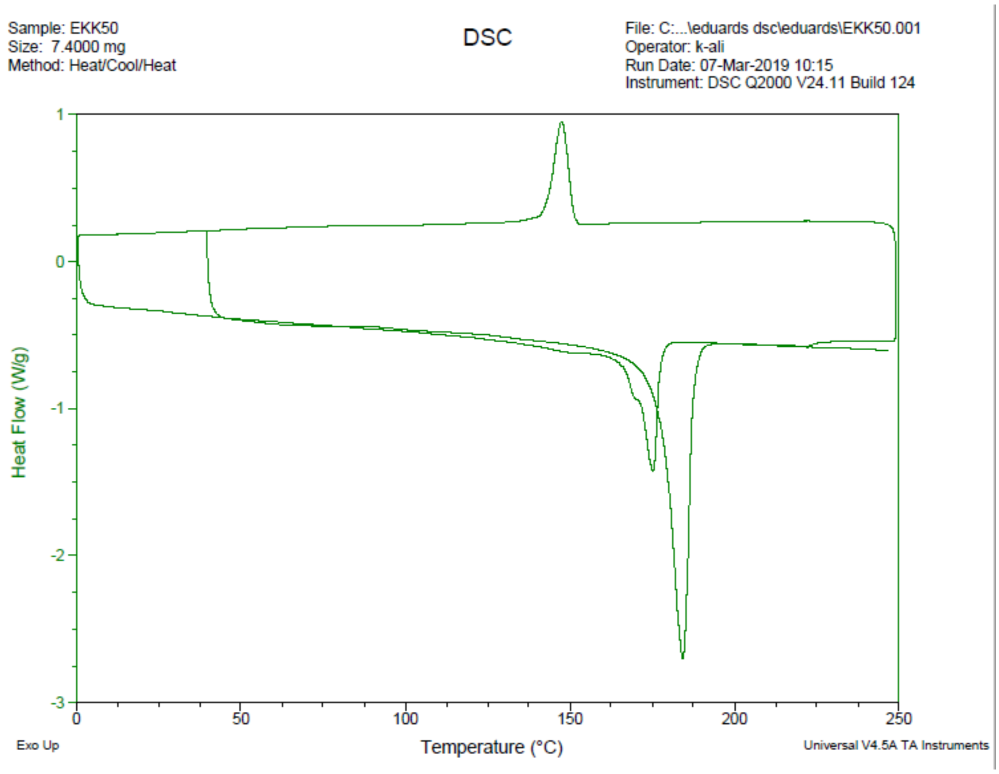

Supplementary Fig 10 - DSC trace of PA-12 coated with p(IBMA-Y1MA) synthesized in a 60 ml autoclave, showing a T_c at ~145-150ºC, a T_m ~175-180ºC (PA-12), and a T_g at 170-175ºC (outer coating composed of P(IBMA-Y1MA), with the T_m and T_g taken from the second heating cycle, and the T_c from the cooling cycle.

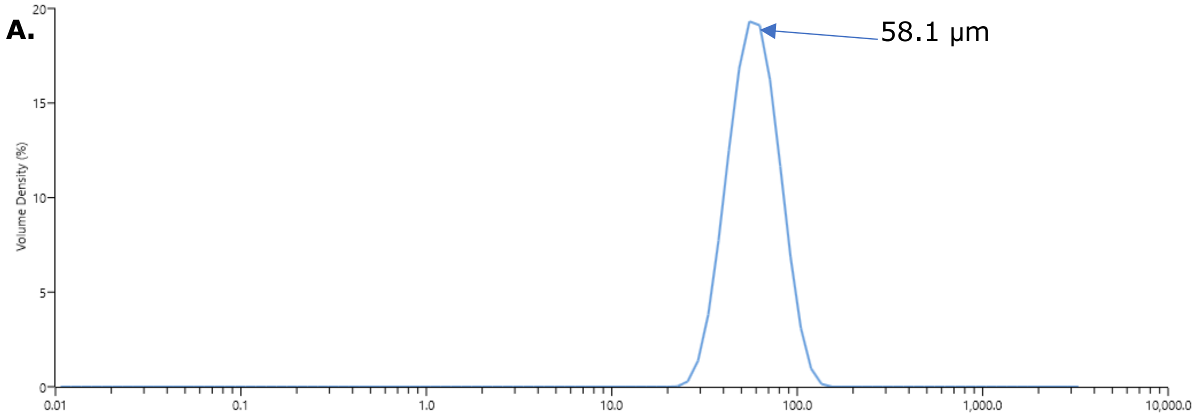

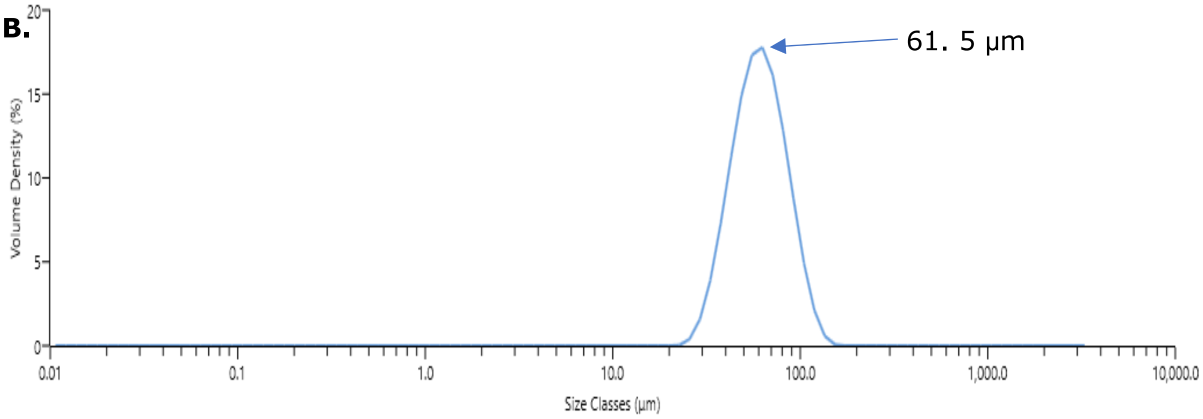

Supplementary Fig 11 – Particle size analysis (LDS) A.) Particle size distribution of uncoated commercial PA-12 particles. The peak indicates that most of the particles are of the size 58.1 µm (± 0.63%). B.) Particle size distribution of PA-12 coated with P(IBMA-Y1MA) particles. The peak indicates that the majority of the particles are of the size of 61.5 µm (± 0.82%).

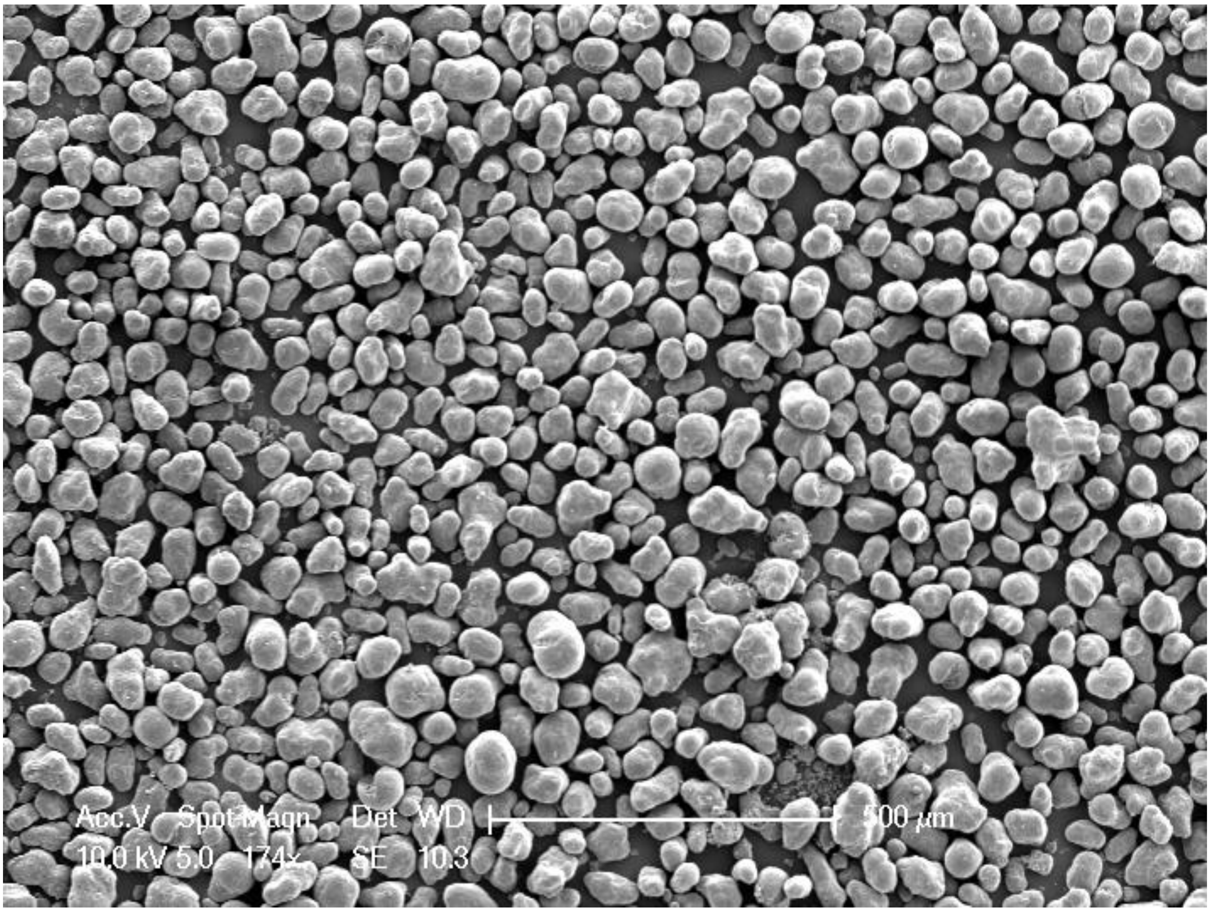

Supplementary Fig 12 – SEM image of PA-12 particles coated with P(IBMA-Y1MA) (Yellow)

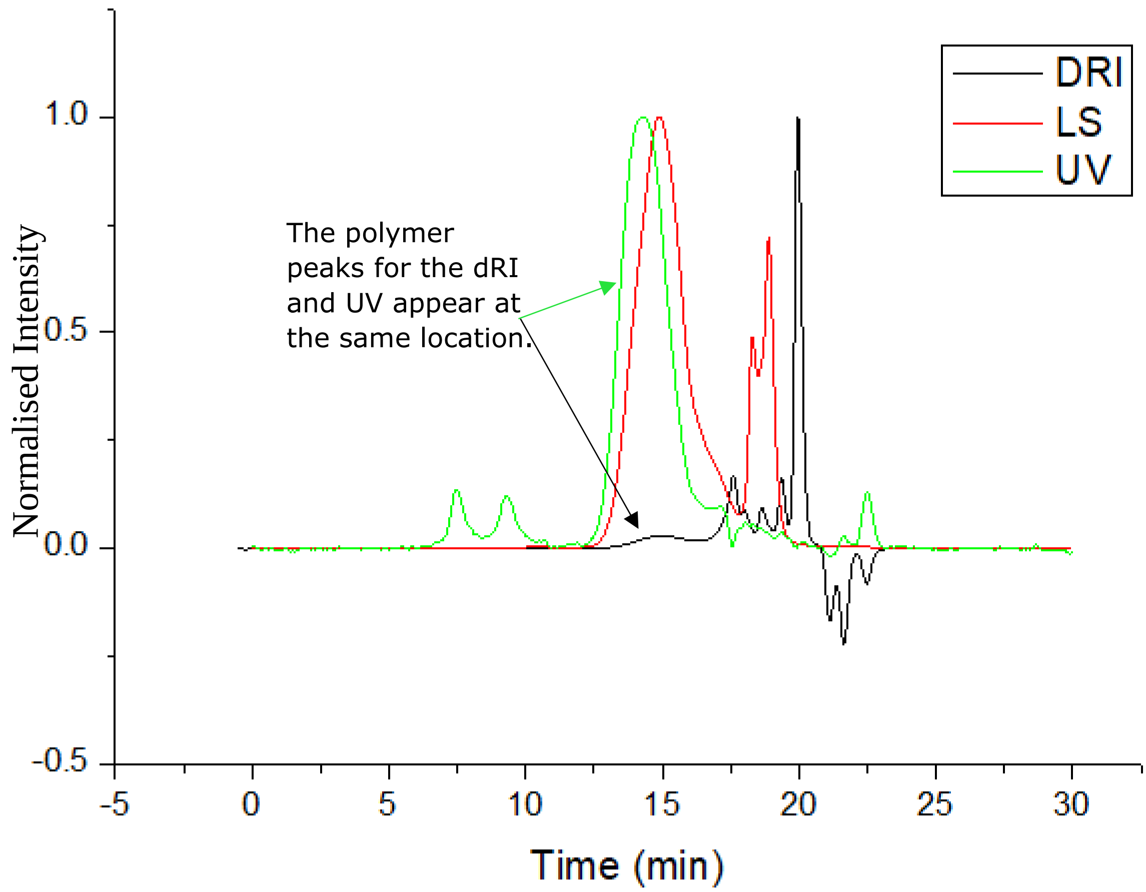

Supplementary Fig 13 - GPC Chromatogram of P(IBMA-DR1MA) outer coating extracted from PA-12 particles coated with P (IBMA-DR1MA) synthesized in a 60 mL autoclave. Showing that UV (ultraviolet) and dRI (differential refractive index) peaks correspond to the same species, demonstrating successful co-polymerisation. LS (light scattering) also has a polymer peak at the same time point.

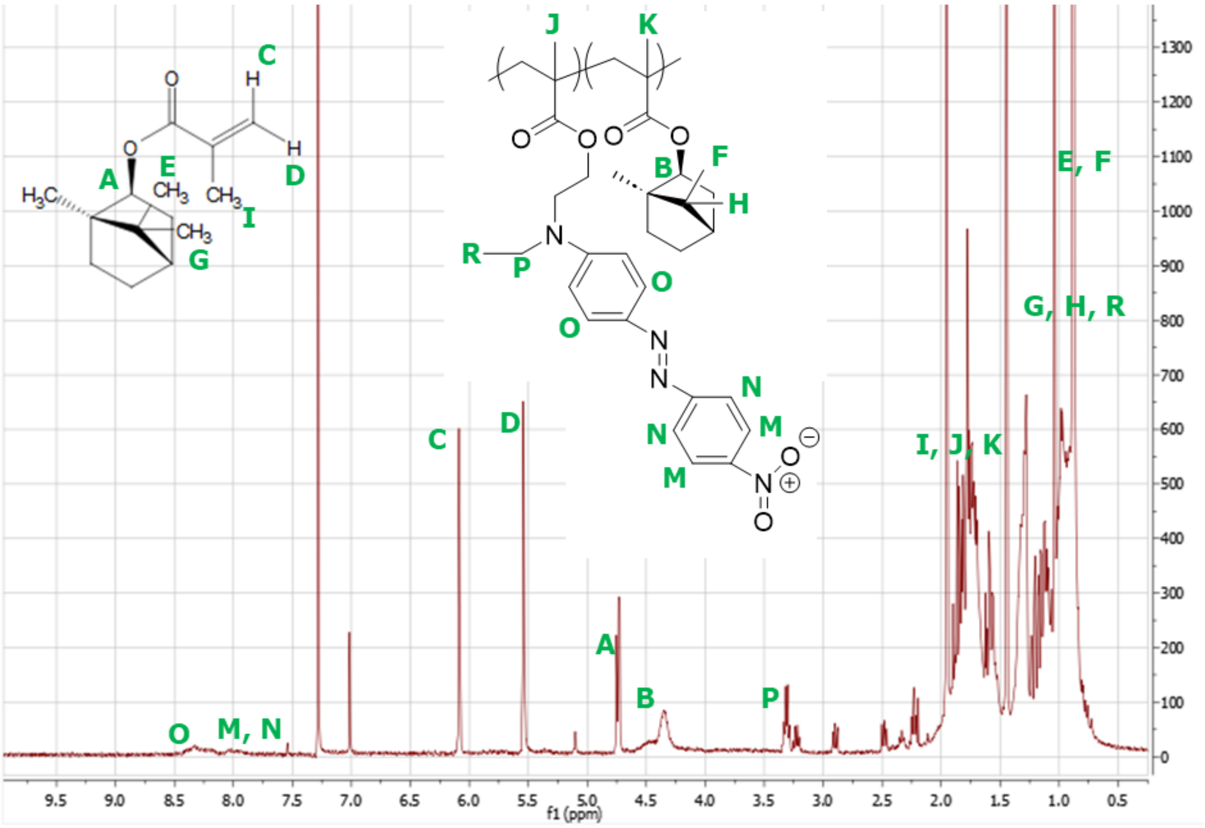

Supplementary Fig 14 – ^{1}H NMR of P(IBMA-DR1MA) in $CDCl_3$ showing residues of IBMA.

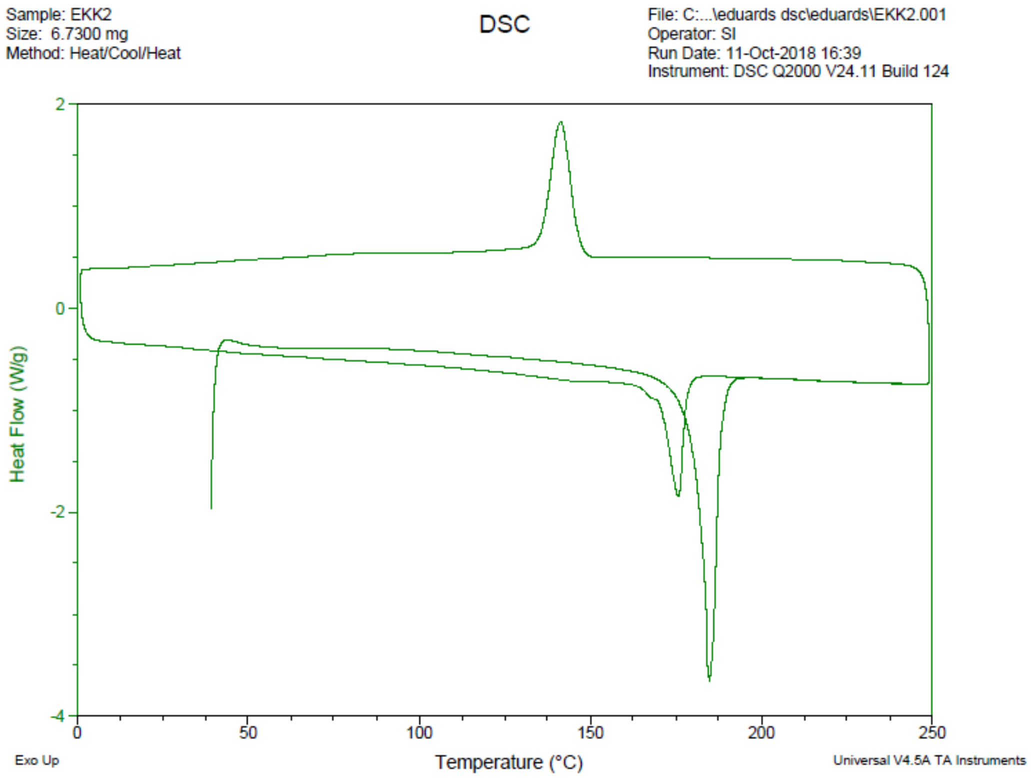

Supplementary Fig 15 - DSC trace of PA-12 coated with p(IBMA-DR1MA) synthesized in a 60 ml autoclave, showing a T_c at ~145-150°C, a T_m ~175-180°C (PA-12), and a T_g at 170-

175ºC (outer coating composed of P(IBMA-DR1MA), with the T_g and T_m taken from the second heating cycle, and the T_c from the cooling cycle.

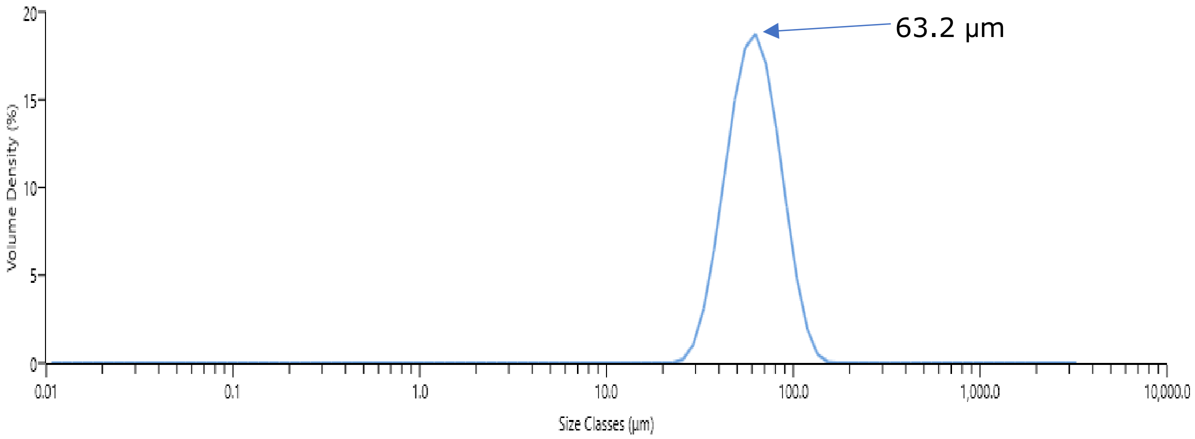

Supplementary Fig 16 – Particle size distribution of PA-12 particles coated with P(IBMA-DR1MA). The peak indicates that most of the particles are of the size of 63.2 µm (± 1.63%), with the average size of uncoated PA-12 being ~58 µm (± 0.63%).

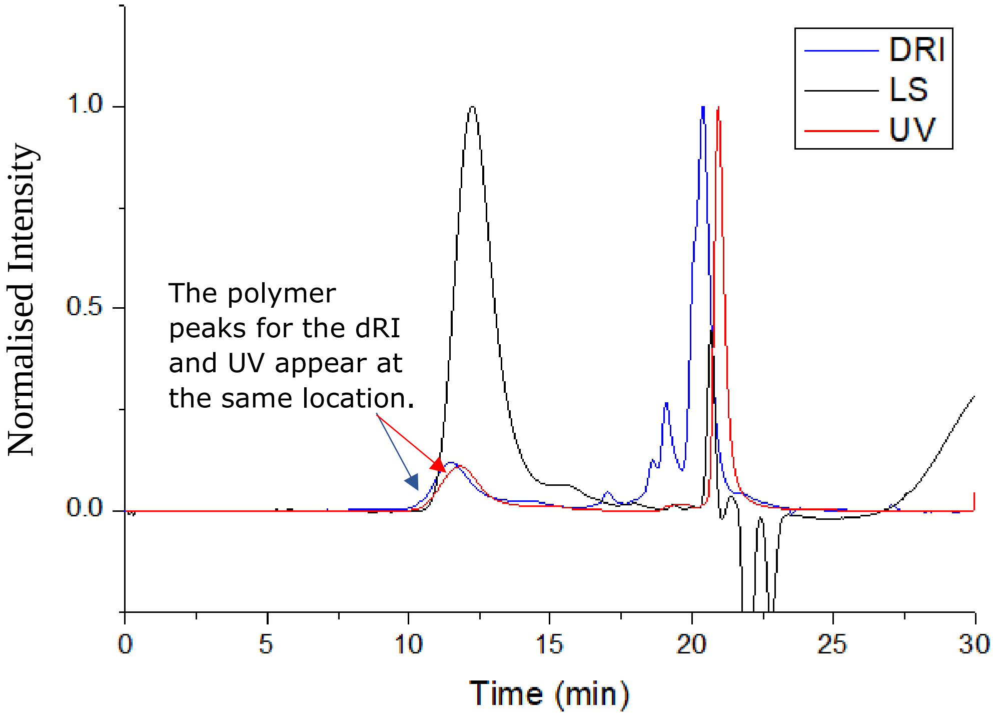

Supplementary Fig 17 – GPC chromatogram of P(IBMA-DB3MA) outer coating extracted from PA-12 particles coated with P (IBMA-DR1MA) synthesized in a 60 mL autoclave. Showing that UV (ultraviolet) and dRI (differential refractive index) peaks correspond to the same species, demonstrating successful co-polymerisation. LS (light scattering) also has a polymer peak at the same timepoint.

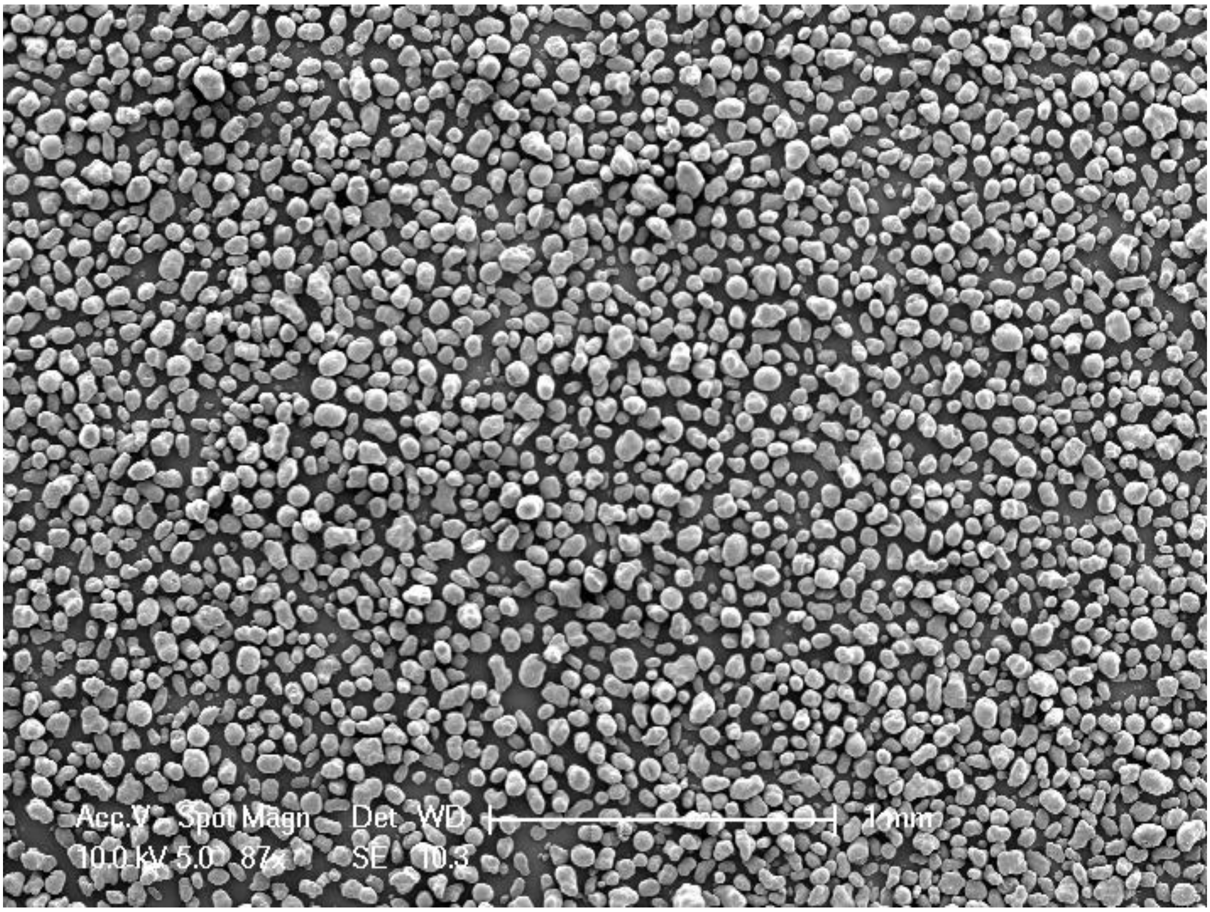

Supplementary Fig 18 – SEM image of PA-12 particles coated with P(IBMA-DR1MA) (Red)

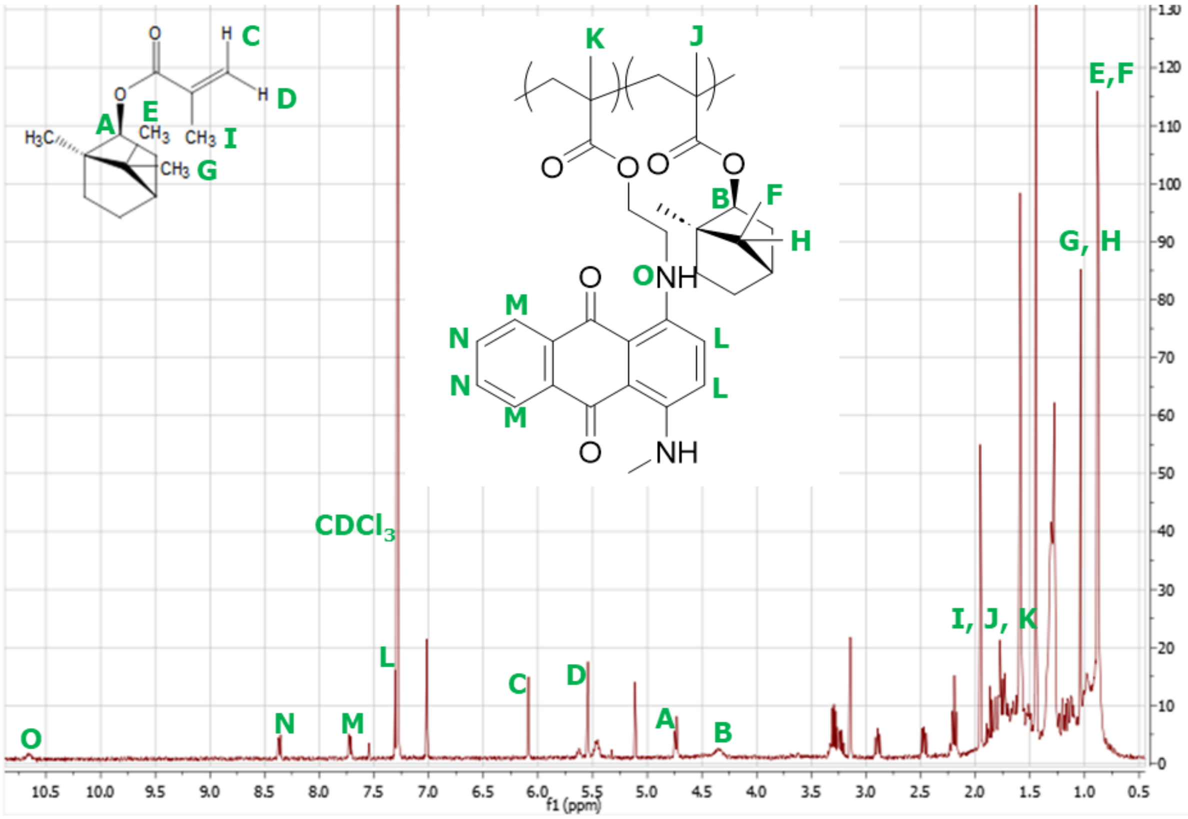

Supplementary Fig 19 – ^{1}H NMR spectrum in $CDCl_3$ showing the formation of P(IBMA-DB3MA) and residues of IBMA.

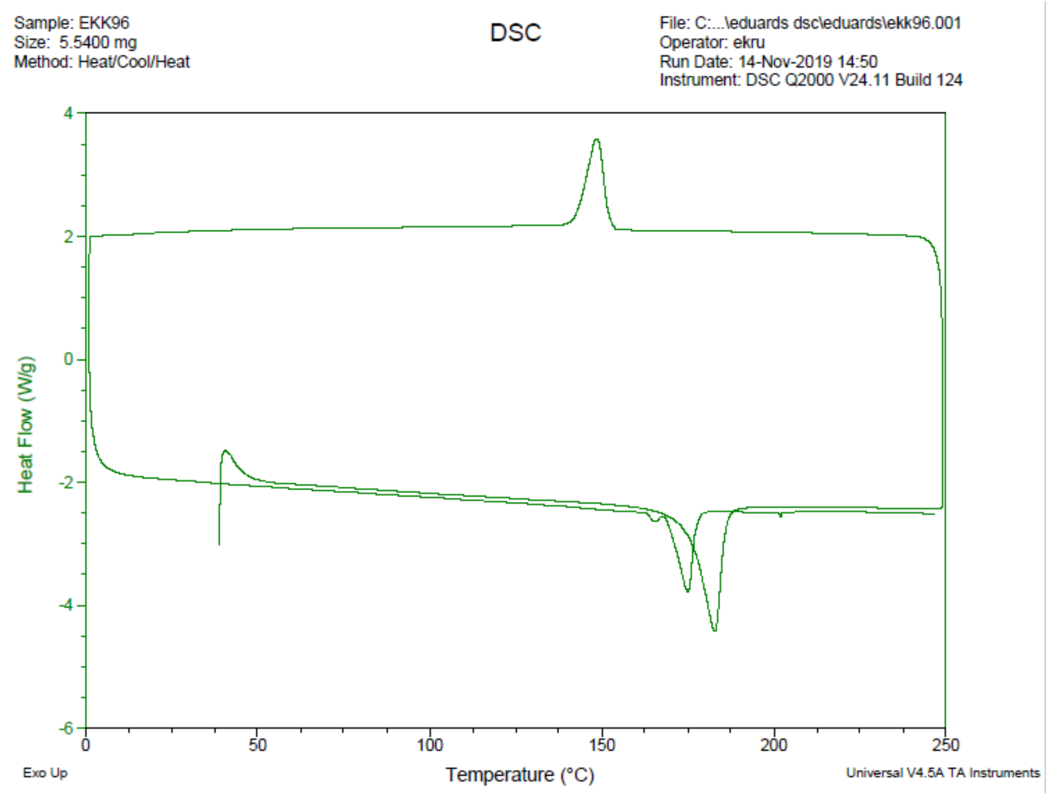

Supplementary Fig 20 - DSC trace of PA-12 coated with p(IBMA-DB3MA) synthesized in a 60 ml autoclave, showing a T_c at ~145-150ºC, a T_m ~175-180ºC (PA-12), and a T_g at 170-

175ºC (outer coating composed of P(IBMA-DB3MA), with the T_g and T_m taken from the second heating cycle, and the T_c from the cooling cycle.

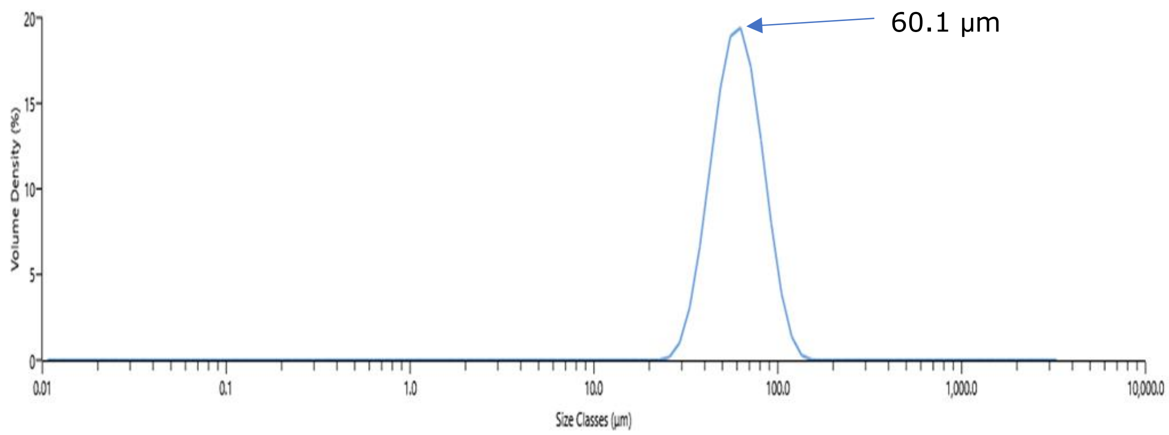

Supplementary Fig 21 –LDS analysis of PA-12 particles coated with P(IBMA-DB3MA) revealed an average particle size of 60.1 µm (± 1.45).

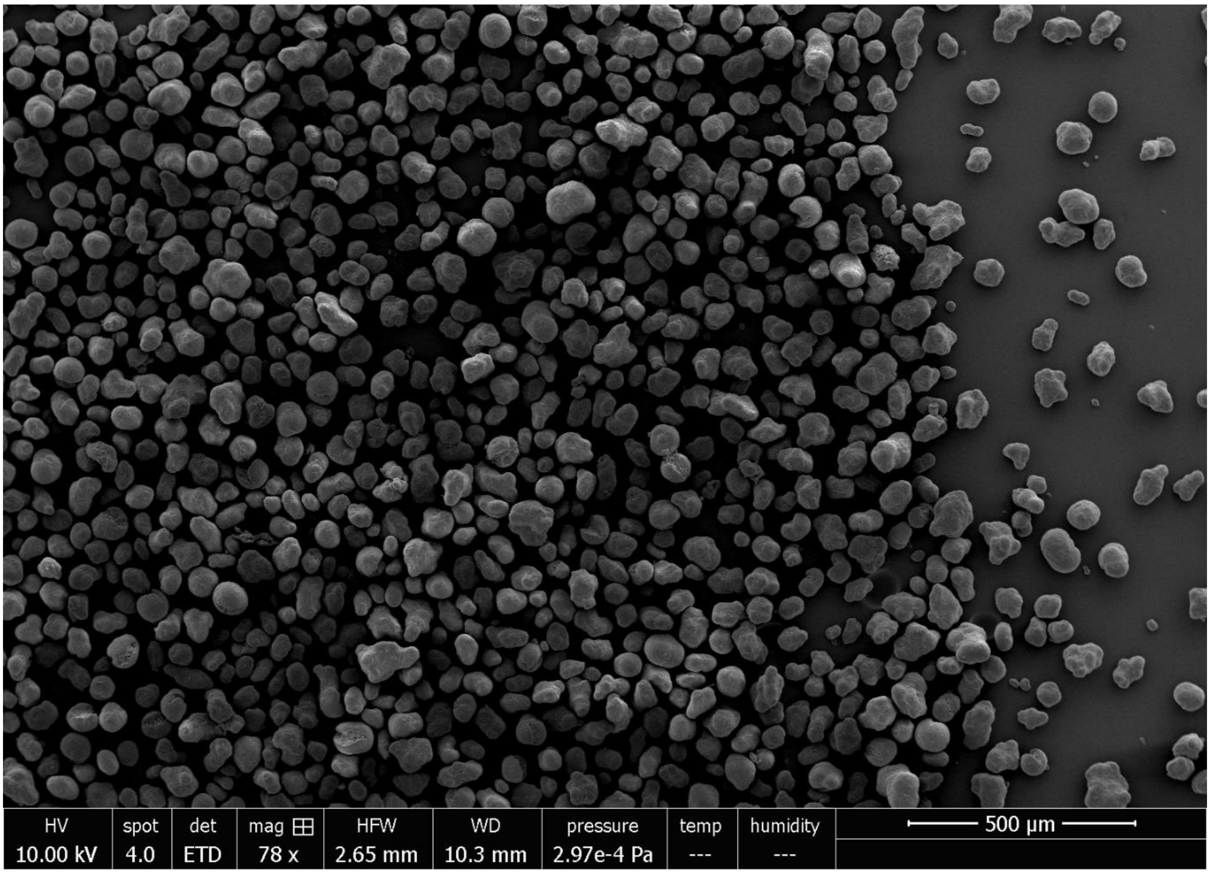

Supplementary Fig 22 – SEM image of PA-12 particles coated with P(IBMA-DB3MA)(Blue)

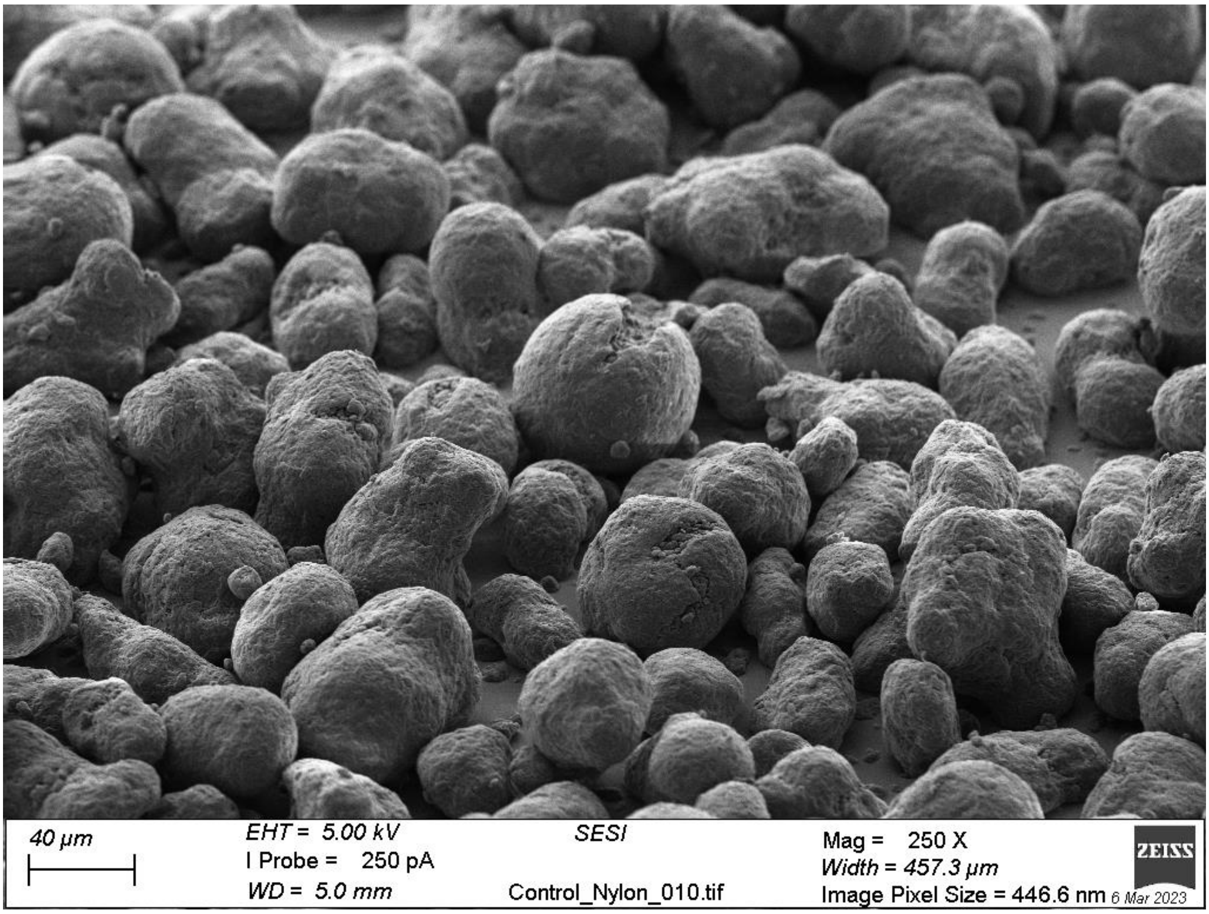

Supplementary Fig 23 - SEM of commercial virgin PA-12 (uncoated) showing the characteristic ‘potato’ shape and the range of particles sizes that are present in the commercial sample.

The SEM images (Supplementary Fig 12, 18, 22, and 23) demonstrate that the commercial PA-12 particles are not significantly changed by our coating process; the SEMs look the same before and after coating. There are smaller particles present initially from the commercial process used to make the (PA-12) and these are not changed significantly after our colour coating process.

Differing loadings of the dye monomers in the copolymers are used, not because of coating efficiency, but because some of the dye monomers have a more intense colour than others.

Scale up (1L) of synthesis of PA-12 particles coated with P(IBMA-dye monomer):
In order to print via SLS with a commercial printer (eg. Formiga P100) at least 600 grams of material are needed to operate the printer. Therefore it was impractical to produce such amounts with a 60 mL autoclave, so the synthesis of PA-12 coated with p(IBMA-dye monomer) was scaled up using a 1 L autoclave. A 1 L autoclave has been previously used for a variety of polymerisations.[1] The results were analogous to that seen in the 60 ml reactions and this was shown through GPC, DSC, and ^{1}H NMR (Supplementary Fig 24-33).

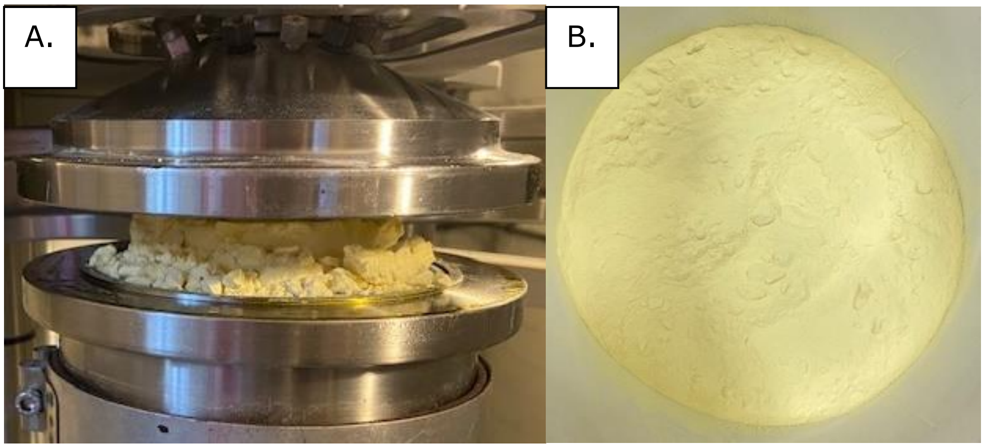

Supplementary Fig 24 – 500 grams of PA-12 coated with P(IBMA-Y1MA) synthesized with a 1L autoclave. A.) PA-12 coated with P(IBMA-Y1MA) being removed from 1L autoclave. B.) 500 g of PA-12 coated with P(IBMA-Y1MA).

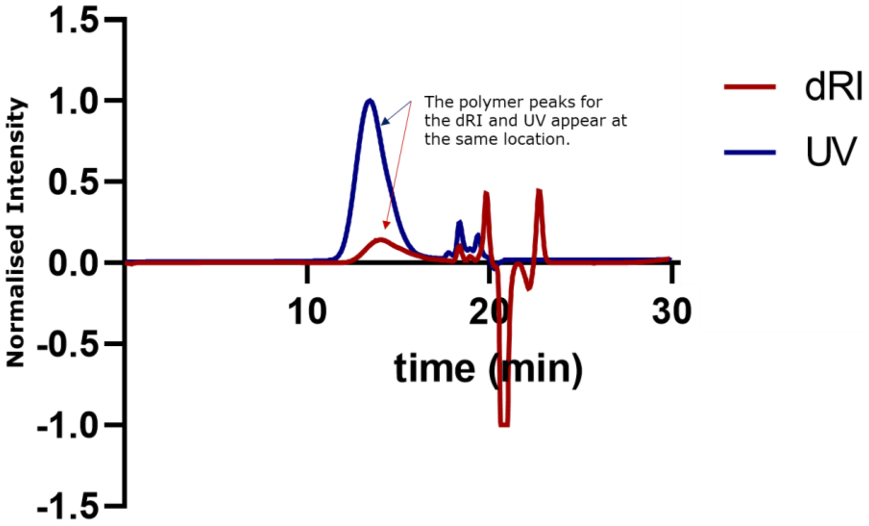

Supplementary Fig 25 - GPC chromatogram of P(IBMA-DR1MA) outer coating extracted from PA-12 particles coated with P (IBMA-DR1MA) synthesized in a 1 L autoclave. Showing that UV (ultraviolet) and dRI (differential refractive index) peaks correspond to the same species, demonstrating successful co-polymerisation.

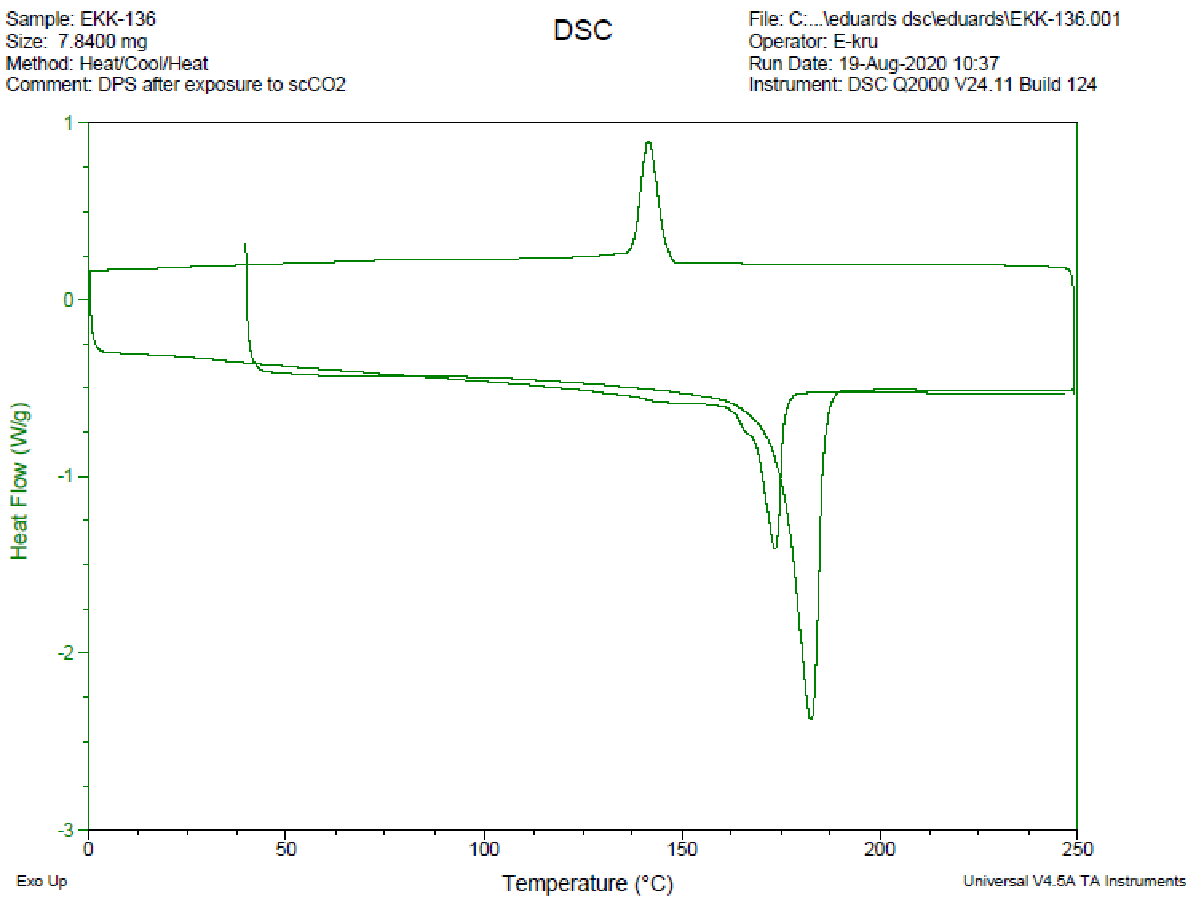

Supplementary Fig 26 - DSC trace of PA-12 coated with p(IBMA-DR1MA) synthesized in a 1 L autoclave, showing a T_c at ~145-150ºC, a T_m ~175-180ºC (PA-12), and a T_g at 170-175ºC (outer coating composed of P(IBMA-DR1MA), with the T_g and T_m taken from the second heating cycle, and the T_c from the cooling cycle.

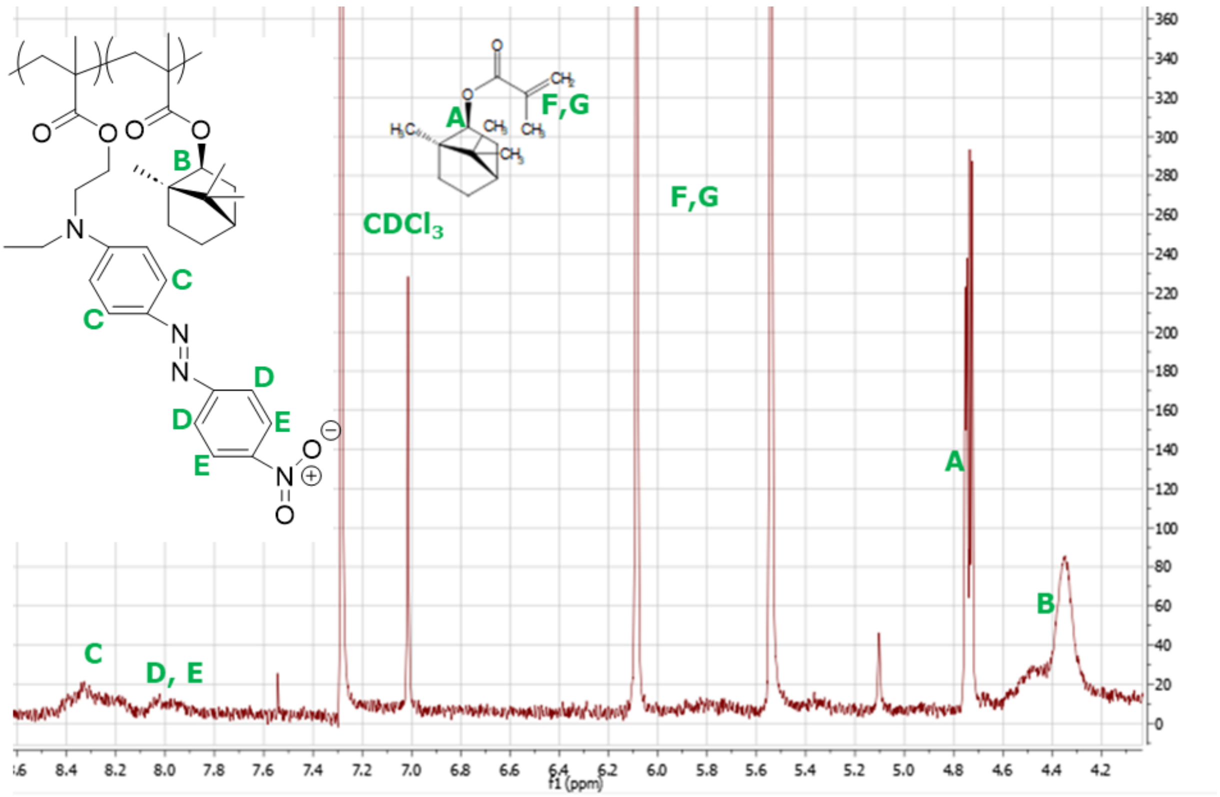

Supplementary Fig 27 - ^{1}H NMR of p(IBMA-DR1MA) outer shell synthesized in 1L autoclave. ^{1}H NMR (400 MHz, $CHCl_3$-d_3, δ): 4.30-4.60 (mp, 1*H*, CH), 7.90-8.10 (mp, 2H, ArH)(mp, 2H, ArH), 8.10-8.40 (mp, 2H, ArH)

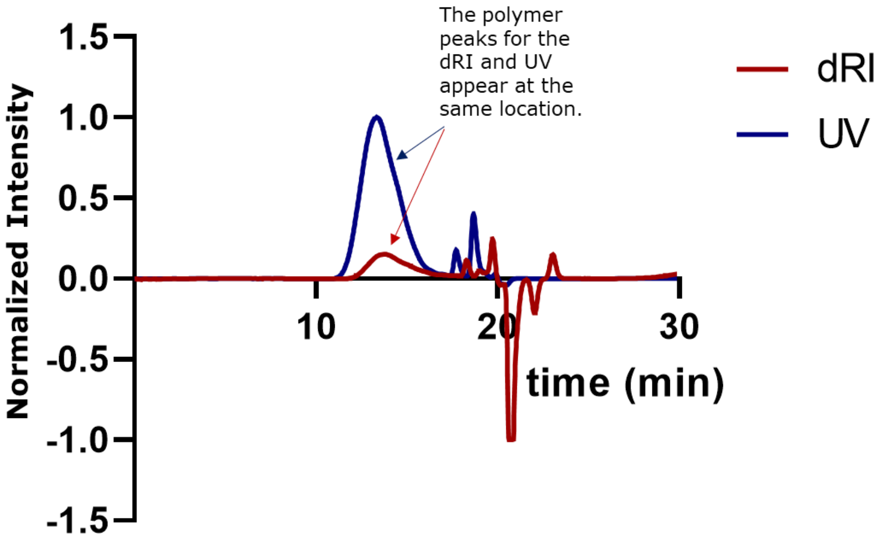

Supplementary Fig 28 - GPC chromatogram of p(IBMA-Y1MA) outer coating extracted from PA-12 particles coated with p(IBMA-Y1MA) synthesized in a 1 L autoclave. Showing that UV (ultraviolet) and dRI (differential refractive index) peaks correspond to the same species, demonstrating successful co-polymerisation.

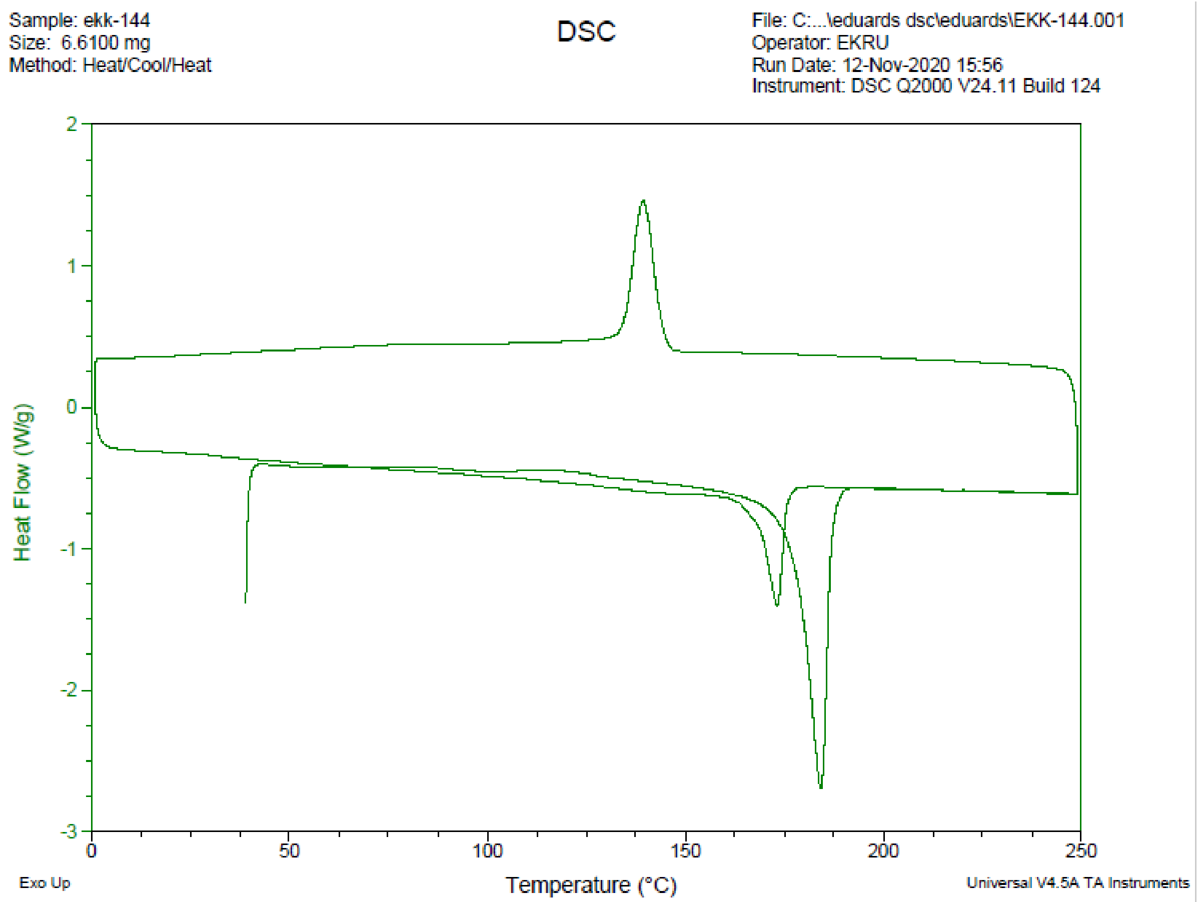

Supplementary Fig 29 - DSC trace of PA-12 coated with p(IBMA-Y1MA) synthesized in a 1 L autoclave, showing a T_c at ~145-150ºC, a T_m ~175-180ºC (PA-12), and a T_g at 170-175ºC (outer coating composed of P(IBMA-Y1MA), with the T_g and T_m taken from the second heating cycle, and the T_c from the cooling cycle.

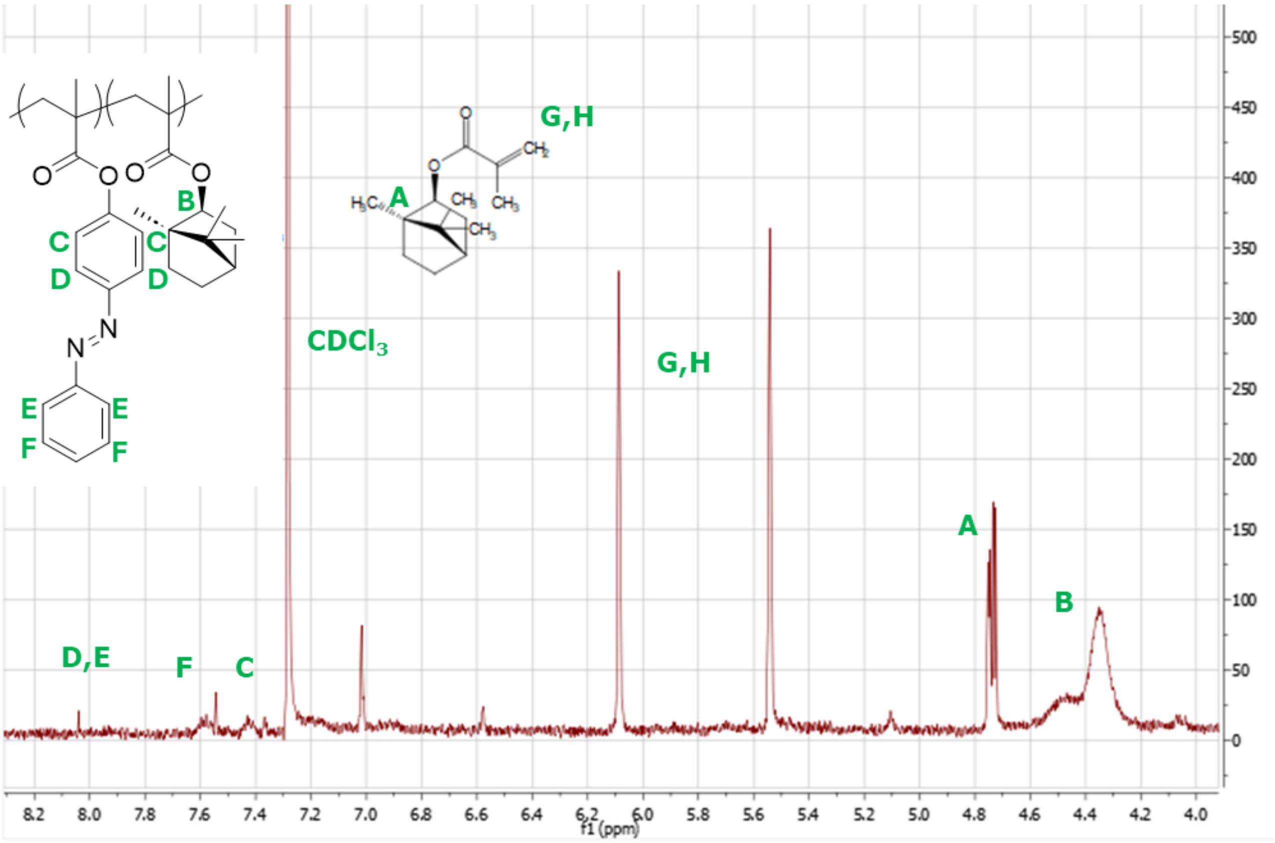

Supplementary Fig 30 - ^{1}H NMR of p(IBMA-Y1MA) outer shell synthesized in 1L autoclave. ^{1}H NMR (400 MHz, $CHCl_3$-d_3, δ): 4.30-4.60 (mp, 1H, CH), 7.39-7.47 (mp, 2H, ArH), 7.48-7.63 (mp, 2H, ArH), 7.80-8.00 (mp, 2H, ArH)(mp, 2H, ArH)

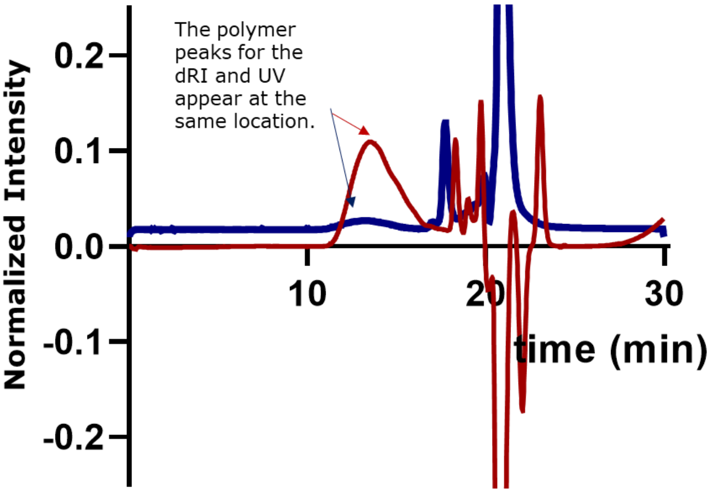

Supplementary Fig 31 - GPC chromatogram of p(IBMA-DB3MA) outer coating extracted from PA-12 particles coated with p(IBMA-DB3MA) synthesized in a 1 L autoclave. Showing that UV (ultraviolet) and dRI (differential refractive index) peaks correspond to the same species, demonstrating successful co-polymerisation.

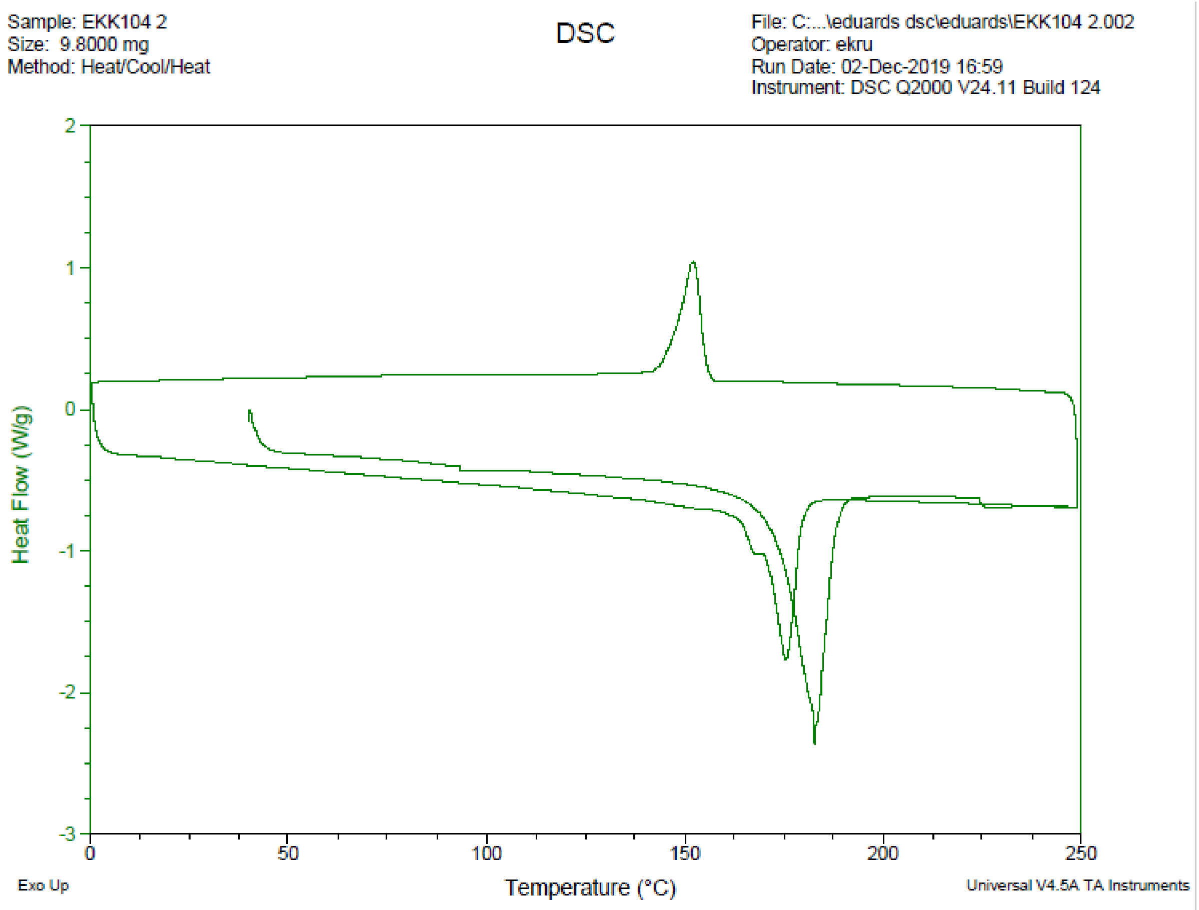

Supplementary Fig 32 - DSC trace of PA-12 coated with p(IBMA-DB3MA) synthesized in a 1 L autoclave, showing a T_c at ~145-150ºC, a T_m ~175-180ºC (PA-12), and a T_g at 170-175ºC (outer coating composed of P(IBMA-DB3MA), with the T_g and T_m taken from the second heating cycle, and the T_c from the cooling cycle.

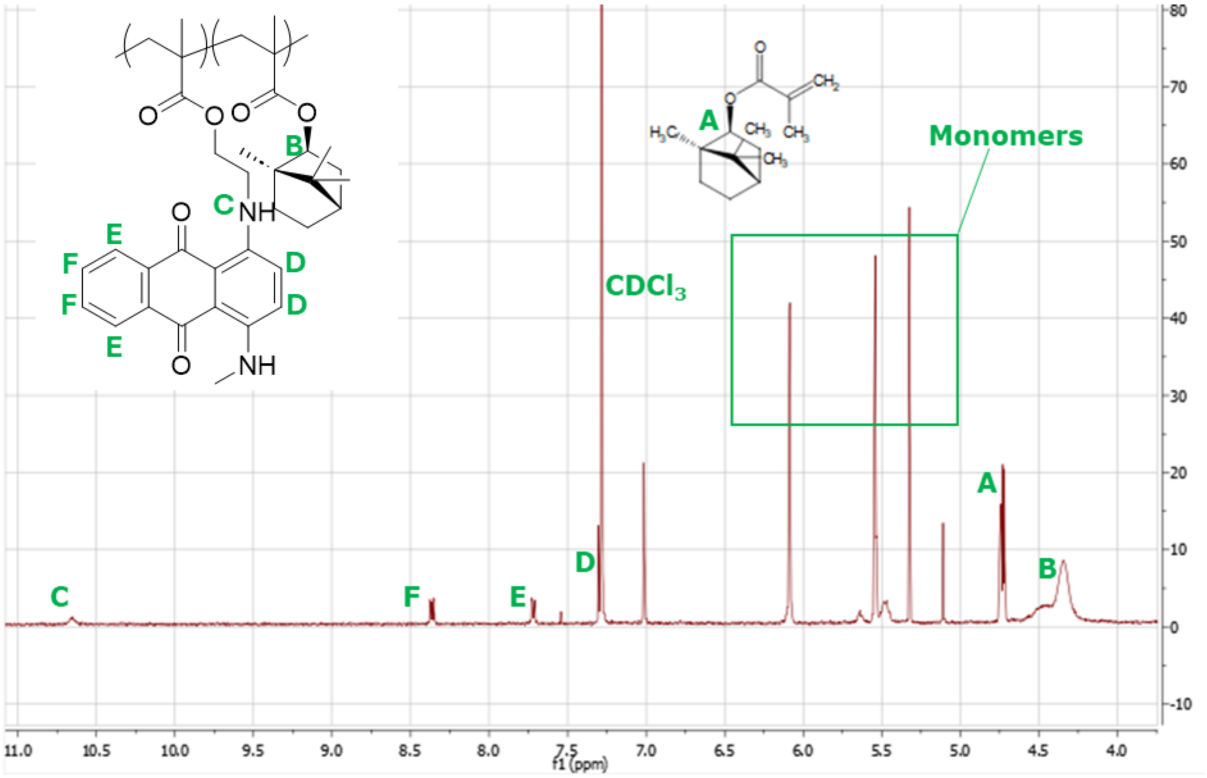

Supplementary Fig 33 - ^{1}H NMR of p(IBMA-DB3MA) outer shell synthesized in 1L autoclave. ^{1}H NMR (400 MHz, $CHCl_3$-*d*$_3$, δ): 4.30-4.60 (mp, 1H, CH), 7.28 (D, 2H, ArH), 7.73-7.75 (mp, 2H, ArH), 8.34-8.38 (mp, 2H, ArH), 10.65 (S, NH)

Cost Analysis for Coating Process

Clearly, new coloured materials for SLS must be commercially viable, so the added cost of the coloured shell must not be significant. The cost of the outer shell needed to be low, so that the cost would be similar to standard PA-12 and lower than what is paid for the current dyeing process. A preliminary cost analysis was done to compare the cost of the novel-coloured PA-12 materials and standard PA-12 (Supplementary Tables 3 and 4). The approximate cost of the raw materials for synthesis of the yellow, cyan, and magenta dye monomers was between £2 – 5 g-1 giving an additional cost for the coloured coating p(IBMA-dye monomer) £0.042-0.175 g-1 (Supplementary Tables 3 and 4), and these are processes that will undoubtedly be lower at scale.

Cost of DB3MA Synthesis			Cost of DR1MA Synthesis			Cost of Y1MA Synthesis		
Reagents	Amount used (g)	Price (£)	Reagents	Amount used (g)	Price (£)	Reagents	Amount used (g)	Price (£)
Disperse Blue 3	4	5.76	Disperse Red 1	3.35	22.38	Yellow 1	5	10.8
Methyl Methacrylate	16	1.06	Methacryloyl Chloride	1.57	1.76	Methacryloyl Chloride	3.71	0.97
Novozyme 435	0.2	19.02	Triethyl amine	2.43	24.52	Triethyl amine	5.78	15.92
Price per gram = 5.17 £			Price Per gram = 4.90 £			Price per gram = 2.12 £		

Supplementary Table 3 – Cost analysis for the synthesis of dye monomers.

Added cost for Blue PA-12		Added cost for Red PA-12		Added cost for Yellow PA-12	
Reagents	Price for producing 150 g (£)	Reagents	Price for producing 150 g (£)	Reagents	Price for producing 150 g (£)
PA-12	NA	PA-12	NA	PA-12	NA
Isobornyl Methacrylate	5.25	Isobornyl Methacrylate	5.25	Isobornyl Methacrylate	5.25
Disperse Blue 3 Methacrylate	2.52	Disperse Red 1 Methacrylate	21.06	Yellow 1 Methacrylate	1.043
AIBN	0.03	AIBN	0.03	AIBN	0.03
Added price per grams = 0.052 £		Added price per grams = 0.175£		Added price per grams = 0.042 £	

Supplementary Table 4 – Cost of coating P2200 PA-12 particles with an outer shell composed of p(IBMA-dye monomer)

Supporting information for Developing a Colour Mixing System for SLS:

Our strategy for colour mixing was to emulate the Cyan, Magenta, Yellow, and Black (CMYK) colour mixing that is prevalent in ink jet paper printers. This is because even though the mixed powders are a solid, when sintered the melt pool is a liquid so the closest analogue for colour mixing is CMYK or subractive colour mixing.

There are known approaches for predicting CMYK colour mixing used in inkjet printers. This has been achieved through a variety of methods such as the compilation of millions of data points recording data of individual formulations or computational methods such as the use of genetic algorithms.

The key reason why we can not use the established methods for the colour mixing exhibited in this work is that the colours can change significantly during the melting and sintering. We found that this results in the colour of a printed red part being different than the colour of the polymeric powder used. This can be clearly seen in the figure below (Supplementary Fig 34)

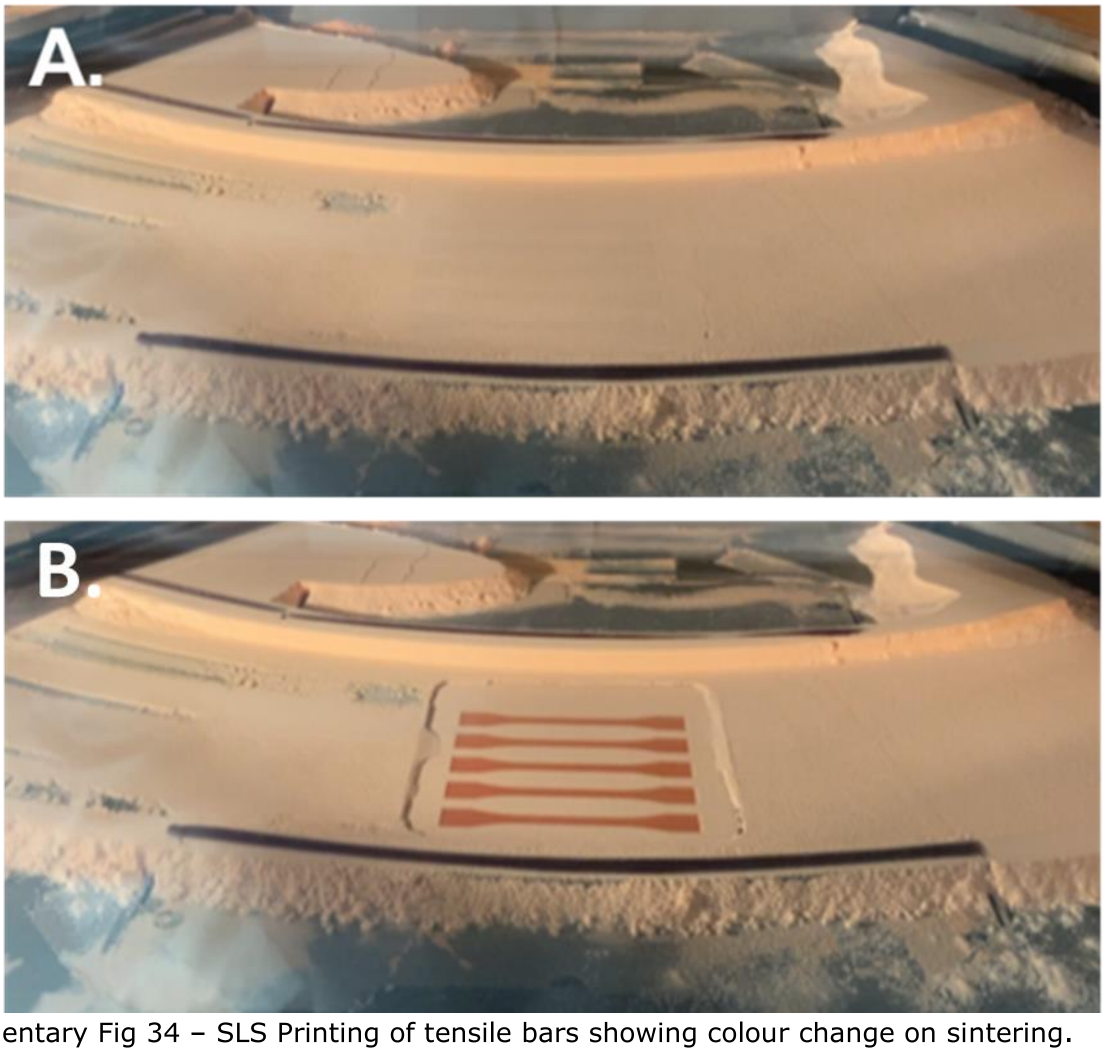

Supplementary Fig 34 – SLS Printing of tensile bars showing colour change on sintering. A. The top layer of powder as it has been spread in this case the 80/20 mix of virgin PA-12 and PA-12 coated with P(IBMA-DR1MA) respectively B. Tensile bars being printed. Note the distinct darkening in the sintered material.

Modelling and Optimisation of Colour Mixture:

Colour Difference Method – Delta E CMC:

The colour difference method allows for the evaluation of the colour difference (ΔE) between two samples, based on the general model (Equation 1).

$$\Delta E = \sqrt{\left(\frac{\Delta L}{l \cdot S_L}\right)^2 + \left(\frac{\Delta C}{c \cdot S_C}\right)^2 + \left(\frac{\Delta H}{S_H}\right)^2} \quad (1)$$

ΔE is a pre-defined two-parameter model (*l* and *c*) widely used to evaluate the colour difference between the reference colour and the desired colour formulated by using a predicted recipe. Equation 1 is typically associated to the Colour Measurement Committee (CMC), typically expressed as CMC(*l*:*c*). When using a lightness weight (*l*) of 2.0 and a chroma weight I of 1.0, Equation 2 is intended for use with acceptability data (CMC(2:1)) whereas for perceptibility, CMC(1:1), *l* and *c* are assumed to be 1 and 1, respectively.

According to Equation 1, CMC colour tolerance is based on CIELAB, accounting for lightness (*L*), chroma (C) and hue (*H*), expressed as differences between sample colour possessing the L_2, a_2, and b_2 values and a reference colour L_1, a_1 and b_1 values, as follows:

$$\Delta L = L_1 - L_2 \quad (2)$$

$$\Delta C = C_1 - C_2 \quad (3)$$

$$\Delta H = \left(\Delta a^2 + \Delta b^2 - \Delta c^2\right)^{\frac{1}{2}} \quad (4)$$

with

$$C_1 = \left(a_1^2 + b_1^2\right)^{\frac{1}{2}} \quad (5)$$

$$C_2 = \left(a_2^2 + b_2^2\right)^{\frac{1}{2}} \quad (6)$$

$$\Delta a = a_1 - a_2 \quad (7)$$

$$\Delta b = b_1 - b_2 \quad (8)$$

The S_L, S_C, and S_H, corresponding to the weighting functions for the lightness, chroma, and hue components, respectively, are strongly dependent on the positions of the sample pair in the CIELAB colour space (Equations 9 to 11).[2]

$$S_L = \begin{cases} 0.511, & \text{if } L_1 < 16 \\ \dfrac{0.0409 \cdot L_1}{1 + 0.01765 \cdot L_1}, & \text{if } L_1 \geq 16 \end{cases} \quad (9)$$

$$S_C = \frac{0.0638 \cdot C_1}{1 + 0.0131 \cdot C_1} + 0.638 \quad (10)$$

$$S_H = S_C\left(F \cdot T + 1 - F\right) \quad (11)$$

where

$$F = \sqrt{\frac{C_1^4}{C_1^4 + 1900}} \quad (12)$$

$$T = \begin{cases} 0.56 + \left|0.2\cos\left(H_1 + 168°\right)\right|, & \text{if } 164° \le H_1 \le 345° \\ 0.36 + \left|0.4\cos\left(H_1 + 35°\right)\right|, & \text{otherwise} \end{cases} \quad (13)$$

$$H_1 = \begin{cases} H, & if\ H \ge 0° \\ H + 360°, & \text{otherwise} \end{cases} \quad (14)$$

with

$$H = \arctan\left(\frac{b_1}{a_1}\right) \quad (15)$$

Regression Model:

A multiple linear regression model, expressed as a third-degree polynomial (Equation 16), can be a useful tool to predict the composition of components in a colour formulation.

$$\Delta E_k^{\mathrm{RM}} = \sum_{i=1}^{m}\beta_i z_i + \sum_{i=1}^{m}\sum_{\substack{j=1 \\ i<j, i\neq j}}^{m}\beta_{ij} z_i z_j + \sum_{i=1}^{m}\sum_{j=1}^{m}\sum_{\substack{k=1 \\ i<j<k, i\neq j\neq k}}^{m}\beta_{ijk} z_i z_j z_k + \sum_{i=1}^{m}\sum_{j=1}^{m}\alpha_{ij} z_i z_j \underset{i<j, i\neq j}{\left(z_i - z_j\right)} + \varepsilon_k \quad (16)$$

where ΔE_k^{RM} represents the dependent variable (response) in the experimental condition k, β_I, β_{ij} and α_{ij} (I = 1, ..., m and j = 1, ..., m, where m stands for the number of components in the colour mixture) are the linear regression coefficients, z_i are the independent variables (colour m) and ε_k is the error associated with each experimental condition k.

In Equation (17), the model response (ΔE_k^{RM}) corresponds to the difference between the predict (desired) colour and reference colour (white). For the development of the cubic regression model, the following hypotheses were presumed true: *i)* polymer particles present a narrow particle size distribution sufficient to melt at the same rate during the selective laser sintering process, and *ii)* polymer microparticles of different colours are uniformly distributed (well mixed), exhibiting similar behaviour as the CMYK colour mixing used by paper inkjet printers. In order to estimate the regression coefficients β_I, β_{ij} and α_{ij}, the least square method was employed, and the function to be minimized is expressed as follows:

$$\Im = \sum_{k=1}^{n}\varepsilon_k^2 = \sum_{k=1}^{n}\left(\Delta E_k^{EXP} - \Delta E_k^{RM}\right)^2 \quad (17)$$

where ΔE_k^{EXP} is the experimental data in the condition k, computed in relation to the reference colour based on Equation 1. It is worth mentioning that the white colour is strategically assumed to be the reference colour (L_1 = 89, a_1 = 5 and b_1 = 4) in order to enhance the regression model ability to predict the true effect of the other colours in the mixture, for instance red, blue and yellow.

Particle Swarm Optimisation:

The optimisation procedure based on the swarm optimization (PSO) has been employed to determine the optimum mixture formulation.[3,4]

The numerical tasks basically consisted of the parameter estimation (colour composition z_i – Equation 16), in such way that the optimization problem can be defined as (Equation 18):

$$\min_{\underline{z}} \ \Im = \left(\Delta E^{\mathrm{RM}} - \Delta E^{\mathrm{Target}}\right)^2 \tag{18}$$

subject to:

$$\sum_{i=1}^{m} z_i = 1 \tag{19}$$

where $\Delta E^{\mathrm{Target}}$ is the desired value of the colour difference computed based on Equation 2, ΔE^{RM} corresponds to cubic regression model (Equation 16) used to evaluate the optimal estimated composition values (z_i), and m is the number of components (colours).

The sequential optimization procedure employed for determination of colour composition based on PSO technique tries to find the best candidates for the cubic regression model (Equation 16) by minimization of Equation 18. Basically, PSO considers that n particles (candidates) moving along a multidimensional search space exchange information with other particles iteratively to find the minimum of the objective function (Equation 18). Equations 20 to 22 are used to compute the velocity (v) and position (x) of the particles in the swarm, which are stochastically updated at each iteration as follows:

$$v_k^{(i+1)} = w^{(i)} v_k^{(i)} + c_1 \lambda \left(x_k^{best} - x_k^{(i)} \right) + c_2 \mu \left(x_{global} - x_k^{(i)} \right) \tag{20}$$

$$x_k^{(i+1)} = x_k^{(i)} + v_k^{(i+1)} \tag{21}$$

$$w^{(i)} = w_{max} - \frac{\left(w_{max} - w_{min} \right)}{1 + e^{\left[-\xi_1 (i - \xi_2) \right]}} \tag{22}$$

where c_1 and c_2 are particle acceleration constants, λ e μ random numbers with uniform distribution in the interval [0, 1], x_k^{best} is the best position of particle k in the swarm, x_{global} corresponds to the best position found considering all particles in the swarm, k denotes the particle and i is related to the iteration number. $v_k^{(i+1)}$ correspond to the velocity and $x_k^{(i+1)}$ is the position of each particle k in the swarm. $W^{(i)}$ is the inertia weight represented as a nonlinear decreasing sigmoidal function. $w_{\max}$ and $w_{\min}$ are the maximum and minimum values of the inertia weight $W^{(i)}$ in the interaction i, respectively, i_{max} is the maximum number of iterations (starting from iteration 1), ξ_1 and ξ_2 are related to i_{max} in the follow way $\xi_1 = 10/i_{max}$ and $\xi_2 = i_{max}/2$.

Performance of the Modelling and Optimisation of Colour Mixture Strategy:

A simplex centroid approach was tested. For a three-component system, the simplex centroid method uses a special cubic instead of a standard cubic like in the simplex lattice (Equation 23).[5]

$$E(Y) = \sum_{i=1}^{q} B_i X_i + \sum_{i=1}^{q} \sum_{i>j}^{q} B_{ij} X_i X_j + \sum_{K=1}^{q} \sum_{j<K}^{q} \sum_{i<j}^{q} B_{ij} K X_i X_j X_K$$

Equation 23 – Special cubic model used in simplex centroid design.[5] (23)

The basic simplex centroid experimental points were used, and the model was made. The r^2 was quite high (0.9550). The estimated effects of the three main components (red, yellow, and blue PA-12 powders) matched experimental observations, as red and blue were of equal importance to the overall colour of the mixture, whilst the yellow was less influential (Supplementary Fig 35).

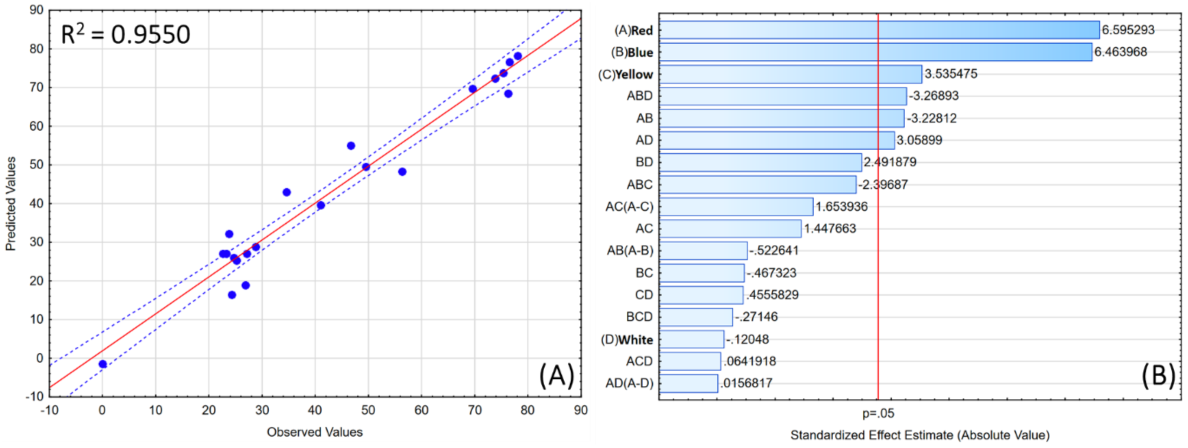

Supplementary Fig 35 – Simplex centroid special cubic model prediction A.) Estimated vs. predicted results B.) Pareto chart for standardized effects with respect to the model variables. (N.B. The prediction model was built based on a 4-factor simplex-centroid design with 21 total runs, of which 19 are unique runs and 2 are replications).

The relationship between the differing main components was further examined through contour mapping of simplex space. The results were positive as all the values relating to the ΔE were between 0 and 100, making this model sound (Supplementary Fig 36).

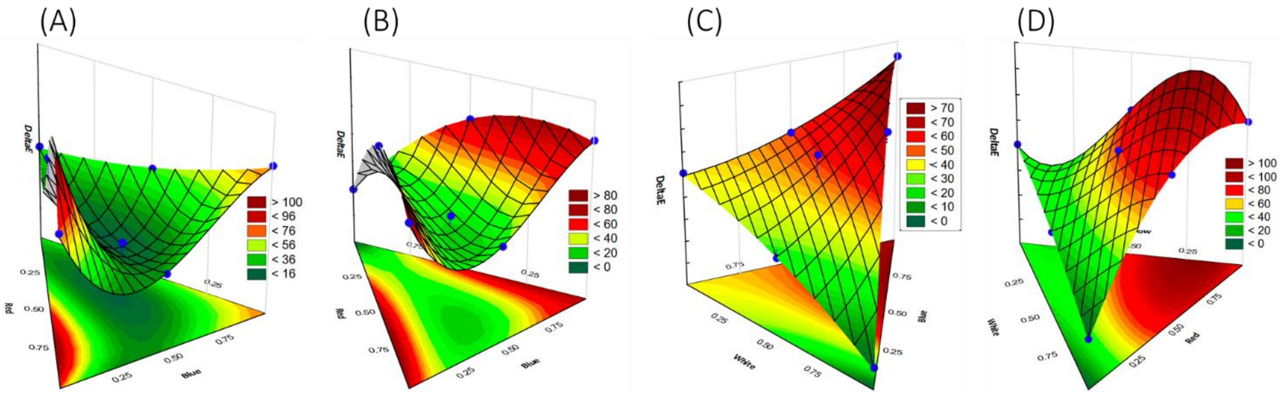

Supplementary Fig 36 – Simplex centroid fitted surface for differing colours A.) Red: Blue: Yellow B.) Red: Blue: White C.) Blue: Yellow: White D.) Red: Yellow: White.

Before printing more colour formulations, a model based on all the data points compiled up until that point was created. This model was made to see if the relationship between the colours-maintained accuracy, even when more data was added. In total, 67 mixtures

were used. The r^2 value was 0.9229, which is lower but deemed acceptable, and the estimated effects of the main colours matched up with what had been seen experimentally. In previous models the red and blue PA-12 inks were the most influential, whilst the yellow powder was about 50 % as influential (Supplementary Fig 37).

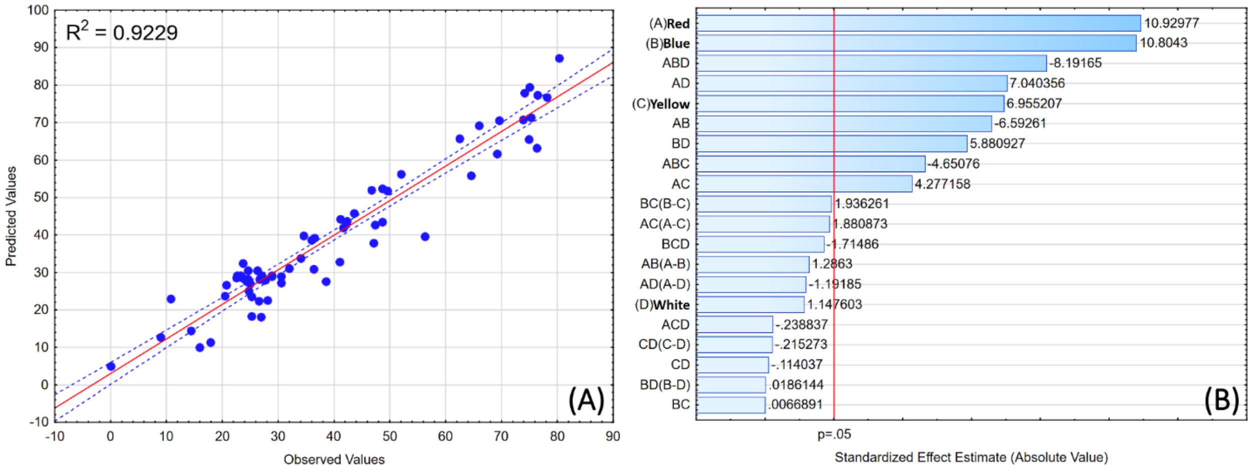

Supplementary Fig 37 – Mixture design (67 points) cubic model prediction. A.) Estimated vs. predicted values B.) Pareto chart for standardized effects with respect to the model variables.

The contour maps show that this type of model could adequately be used to predict the formulation of target colours as the values on the fitted curves were between 0 and 100 (Supplementary Fig 38).

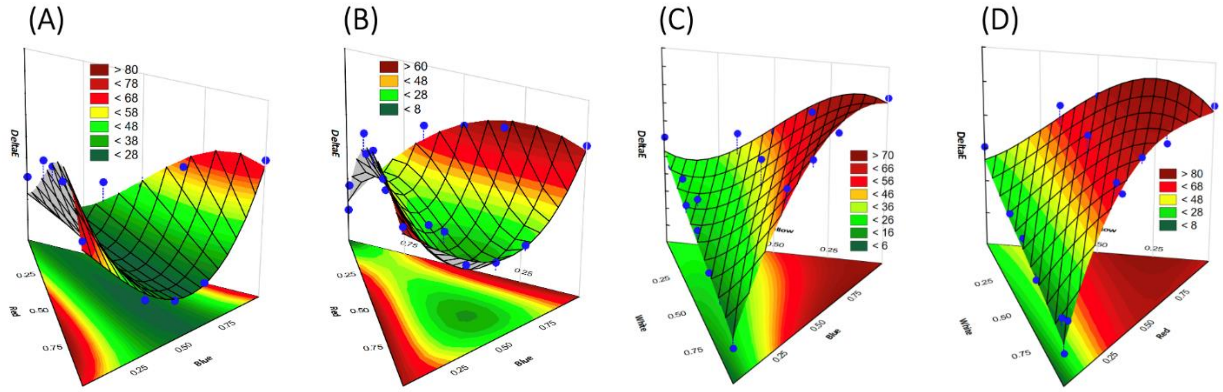

Supplementary Fig 38 – Mixture design (67 points) fitted surface for the different colours. A.) Red: Blue: Yellow B.) Red: Blue: White C.) Blue: Yellow: White D.) Red: Yellow: White.

From this point, the data gathered from this model was fed into a particle swarm optimizer to yield predicted formulations with the four component 'inks' (red, blue, yellow, and white). The combination of these two techniques would rapidly produce estimated formulations for target colours. The formulations for nine target colours were predicted with the simplex centroid special cubic model plus particle swarm optimizer. In these nine colours, three of them were green, two were blue, two were red, and two were brown (Supplementary Table 5).

Colour Target	Colour fraction (wt)				Results			
	Blue	Red	Yellow	White	L	A	B	Colour
Green	0.0626	0.0000	0.6418	0.2956	62	-13	5	green
Green	0.0987	0.0653	0.6055	0.2306	45	6	16	brown
Green	0.0877	0.0133	0.6255	0.2735	57	-6	6	green
Blue	0.7033	0.2333	0.0604	0.0030	19	5	-12	violet
Blue	0.6609	0.1550	0.1587	0.0255	22	-3	-13	Blue
Red	0.0574	0.8409	0.0714	0.3030	32	61	50	red
Red	0.0553	0.8006	0.0848	0.0594	30	50	48	red
Brown	0.2786	0.2917	0.0000	0.4297	27	10	15	brown
Brown	0.1428	0.5783	0.1070	0.1719	24	31	31	orange

Supplementary Table 5 – Results from predicted formulations for the target colour of SLS printed parts.

From Supplementary Table 5, it can be seen that several of the formulations were predicted correctly as two out of the three green targets were green. The blue and brown targets both correctly predicted one out of two of the targets. The red target also yielded positive results and both of the predicted formulations produced red parts. The results can be viewed in a positive light as the formulations that missed, resulted in colours similar to that of the targets. This is evident as the formulation that failed to make blue yielded a violet part, and the brown formulation gave an orange part; these misses are quite close in colour space to the desired target. The model could be improved through a simple methods, such as boundary conditions being introduced into the particle swarm optimization method, which would automatically remove certain predicted formulations. The modelling process with the combined simplex centroid plus particle swarm optimization yielded other notable observations.

From the formulations predicted by the model, it was seen that a variety of different formulations could potentially be used to produce the same target colour. This could have a beneficial impact on the industrial appeal of the project, as this could serve as a cost minimization tool. As is widely known, different paints cost varying amounts, as the ingredients to produce them can vary. The same applies in this project as each of the coloured PA-12 ‘inks’ are more expensive than standard PA-12, as there is cost from the dye monomers and IBMA, and each of the dye monomers differ in value as well. This makes the use of the white component (standard PA-12) in formulations appealing as it can lower the cost whilst giving the same colour. By using this method, several formulations could be tested in ordered to determine the best compromise between results and cost. This could also be improved by adding the boundary conditions. Supplementary Table 6 shows an example output of possible formulations for a target colour (L=60, a=10, b=10). In order to achieve the most cost-effective formulation we would avoid the formulations with the highest percentages of red (formulation 2, Supplementary Table 6) as red PA-12 is the most expensive component. The choice would be between the mixes that give the best colour and the least expensive formulation, which would be the formulation with the highest amount of white, but to make a bright and vivid colour the appropriate amount of red, blue, and yellow must be used.

Formulation for Target colour (LAB: 60, 10, 10)	Amount of red (%)	Amount of blue (%)	Amount of yellow (%)	Amount of white (%)
1	0.0014	0.0088	0.2191	0.7707
2	0.0291	0	0	0.9709
3	0.0023	0	0.2479	0.7498
4	0.0134	0	0.1466	0.8401
5	0.0135	0.0242	0	0.9623
6	0.0219	0	0.0665	0.9116
7	0.0139	0.0237	0	0.9624
8	0	0.0223	0.1587	0.819
9	0	0.012	0.217	0.771
10	0.0157	0.0212	0	0.9631
11	0.0059	0.034	0	0.9601
12	0.0247	0.0074	0	0.9679

Supplementary Table 6 **–** Example of output table showing all the possible formulations for just one target colour. The example colour used as LAB values of 60, 10, 10 respectively. This could be used as a cost-optimization tool.

Supporting information for printing of multi-material parts:

Multimaterial parts were printed to prove that parts could be made out of more than one materials as long as the thermal properties of the materials were similar and an appropriate powder delivery system was used. To produce multimaterial parts a mask method was employed which would keep the building part in place during printing, with this method a block was printed that was composed of a bottom red layer, a middle white layer and a top red layer (Supplementary Fig 39B-D). Utilizing this method, a well-known science fiction character was built with a bottom white layer and a top black layer, showing that more complicated parts could be built with this method (Supplementary Fig 39E).

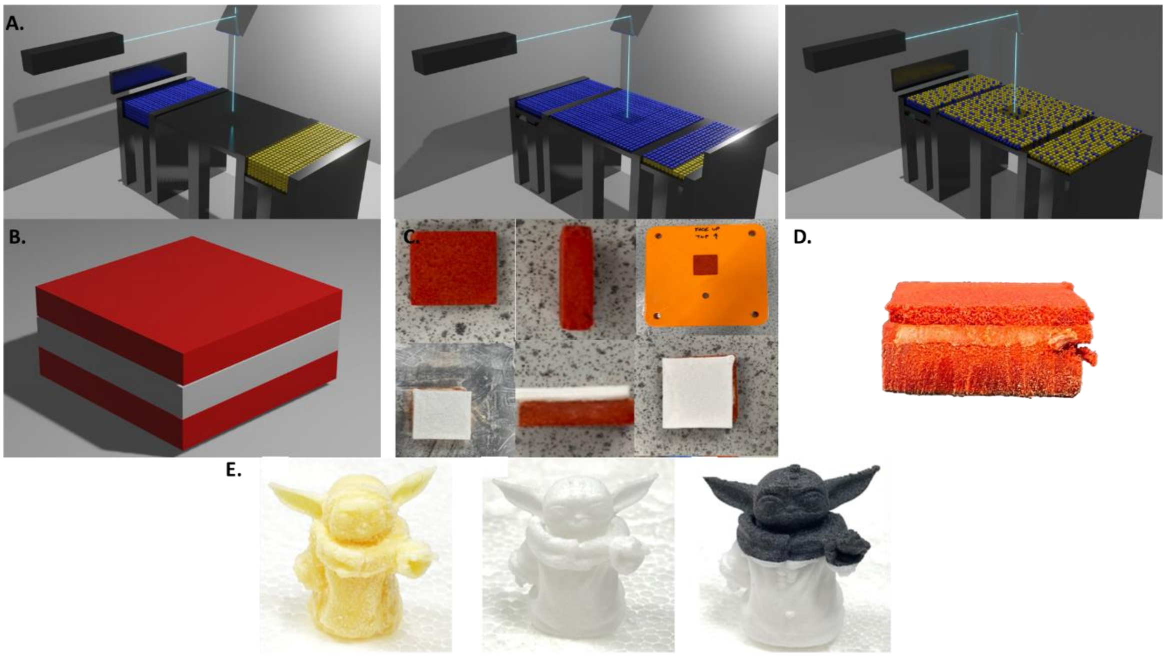

Supplementary Fig 39 – Development of multimaterial SLS. A.) Current powder deposition method resulting in powder mixture, thereby the resulting part would not be made of two distinct materials but would effectively be a blend. B.) Model of desired multimaterial part, block with a bottom composed of red PA-12, middle segment composed of white PA-12, and top composed of red PA-12. C.) Layer by layer production of 3 layered block structure using mask method. D.) Resulting three layered block composed of a red bottom, white middle, and red top. E.) Comparison of different science fiction characters built with yellow PA-12 (left), white PA-12 (middle), and multimaterial white bottom and black top (right).

Supporting information for synthesis and further characterisation of biofilm preventing materials:

PA-12 was coated with six different copolymeric coatings in an attempt to produce anti-attachment materials for SLS. These materials were produced via an analogous method to the one used to produce coloured PA-12 particles, but instead of the dye monomer, biologically active (meth)acrylates were used resulting in six coatings:

- p(IBMA-Bornyl methacrylate)
- P(IBMA-Cinnamyl methacrylate)
- p(IBMA-Myrtenol methacrylate)
- p(IBMA-Neryl methacrylate)
- p(IBMA-modified oleic acid acrylate)
- p(IBMA-modified lactic acid acrylate)

These coated materials were analysed via ^{1}H NMR, GPC, and DSC, much like what was reported for the coloured PA-12 materials (Supplementary Fig 40-45).

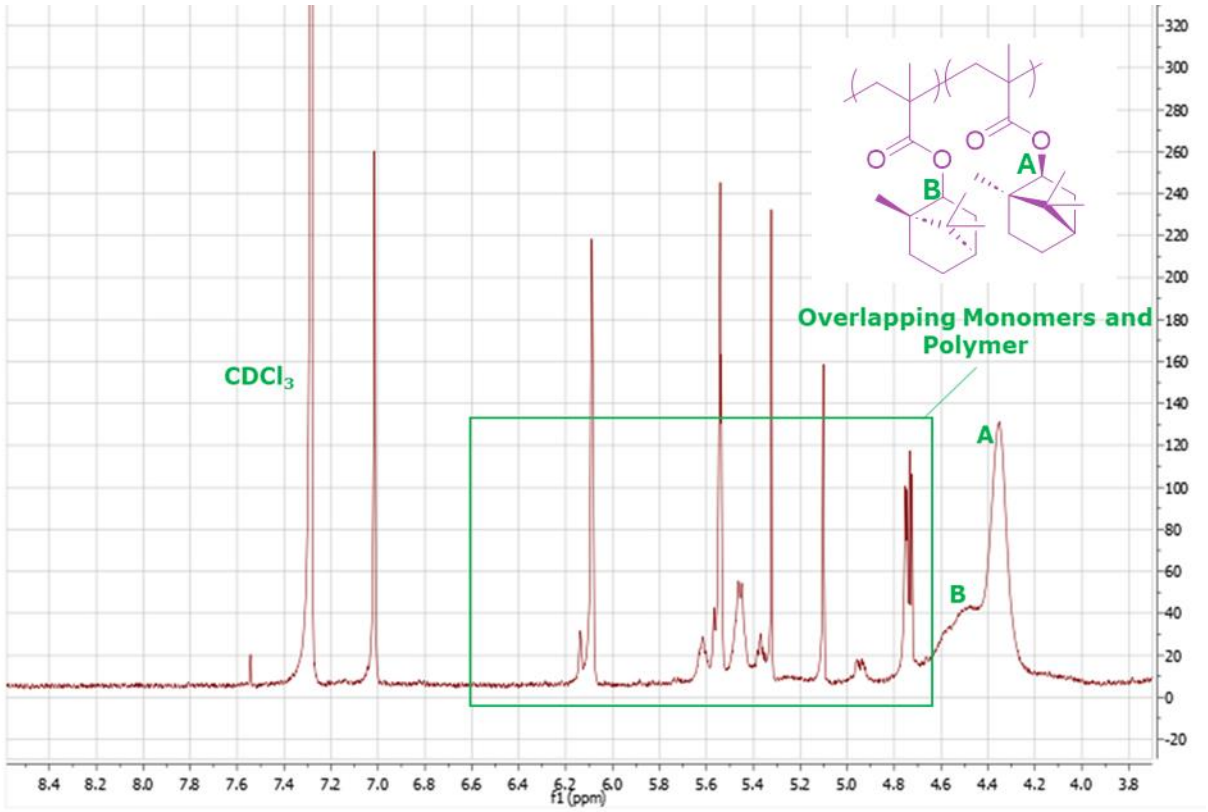

Supplementary Fig 40 - ^{1}H NMR of p(IBMA-Bornyl methacrylate) outer shell synthesized in 60 mL autoclave ^{1}H NMR (400 MHz, $CHCl_3$-d_3, δ): 4.23-4.69 (mp, 1H, CH), 4.23-4.69 (mp, 1H, CH)

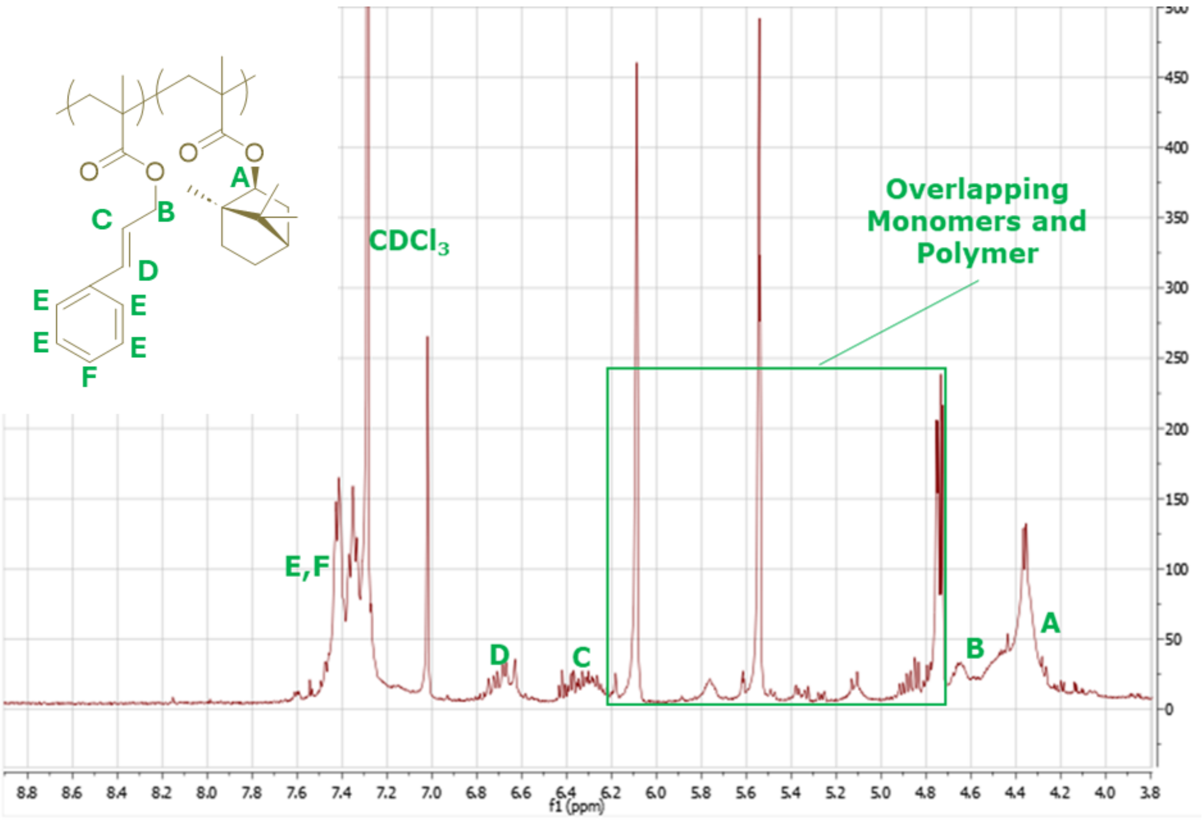

Supplementary Fig 41 - ^{1}H NMR of p(IBMA-Cinnamyl methacrylate) outer shell synthesized in 60 mL autoclave ^{1}H NMR (400 MHz, $CHCl_3$-d_3, δ): 4.23-4.60 (mp, 1H, CH), 4.59-4.70 (mp, 2H, CH_2), 6.20-6.45 (mp, 1H, CH), 6.54-6.83 (mp, 1H, CH), 7.21-7.54 (mp, 2H, ArH) (mp, 2H, ArH) (mp, 2H, ArH) (mp, 2H, ArH) (mp, 2H, ArH)

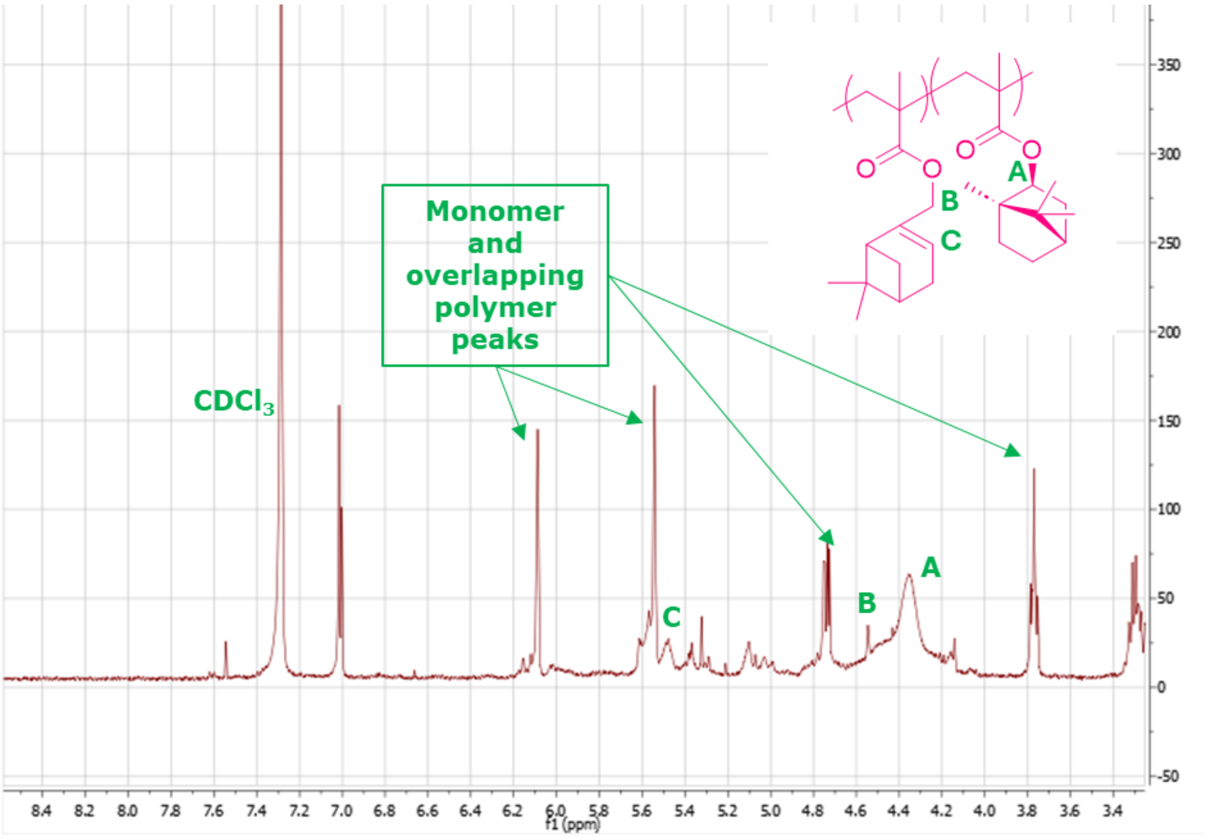

Supplementary Fig 42 - ^{1}H NMR of p(IBMA-Myrtenol methacrylate) outer shell synthesized in 60 mL autoclave ^{1}H NMR (400 MHz, $CHCl_3$-d_3, δ): 4.21-4.63 (mp, 1H, CH), 4.23-4.62 (mp, 2H, CH_2), 5.43-5.51 (mp, 1H, CH)

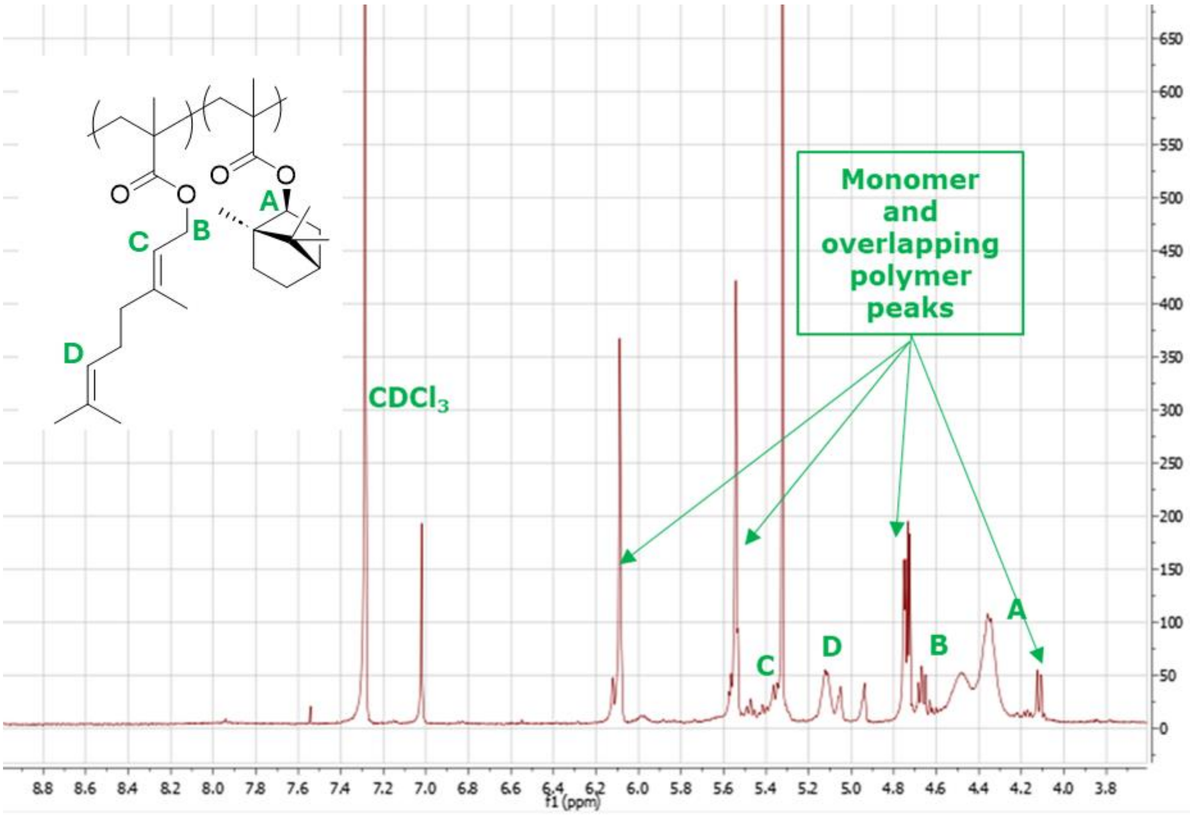

Supplementary Fig 43 - ^{1}H NMR of p(IBMA-Neryl methacrylate) outer shell synthesized in 60 mL autoclave ^{1}H NMR (400 MHz, $CHCl_3$-d_3, δ): 4.23-4.60 (mp, 1H, CH), 4.59-4.70 (mp, 2H, CH_2), 5.02-5.19 (mp, 1H, CH), 5.25-5.43 (mp, 1H, CH)

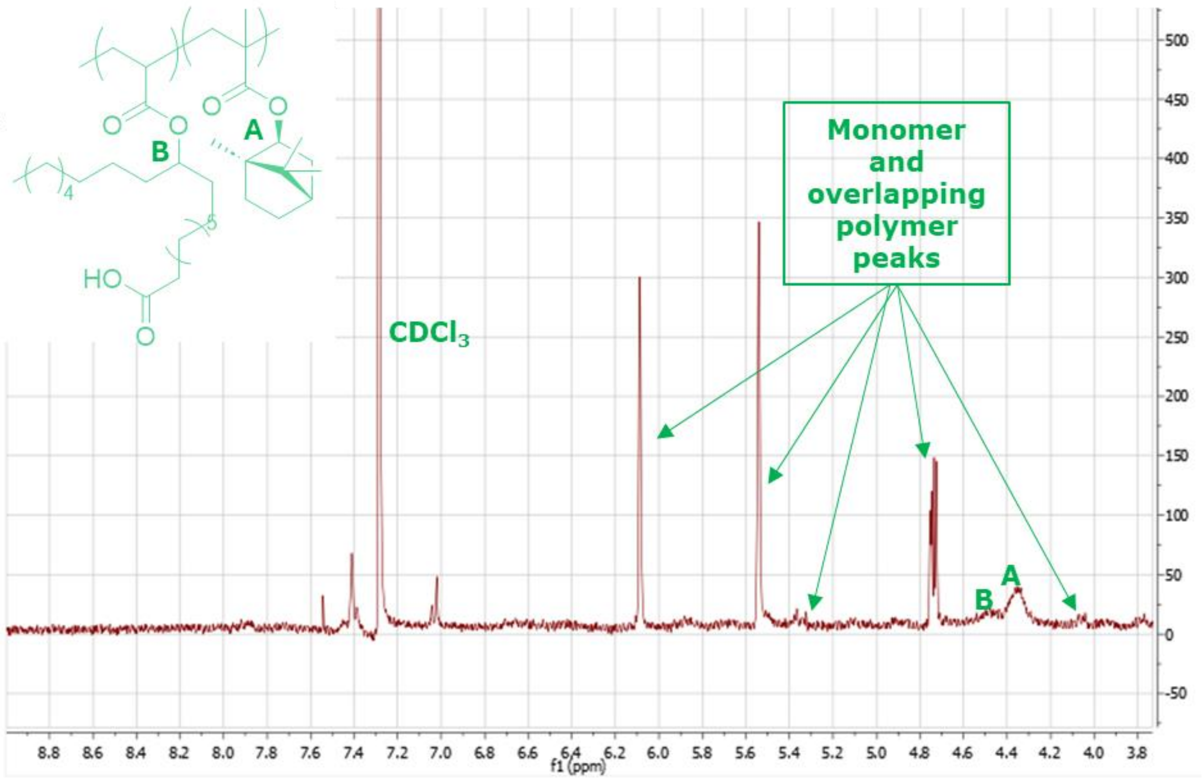

Supplementary Fig 44 - ^{1}H NMR of p(IBMA- modified oleic acid acrylate) outer shell synthesized in 60 mL autoclave ^{1}H NMR (400 MHz, $CHCl_3$-d_3, δ): 4.23-4.60 (mp, 1H, CH)

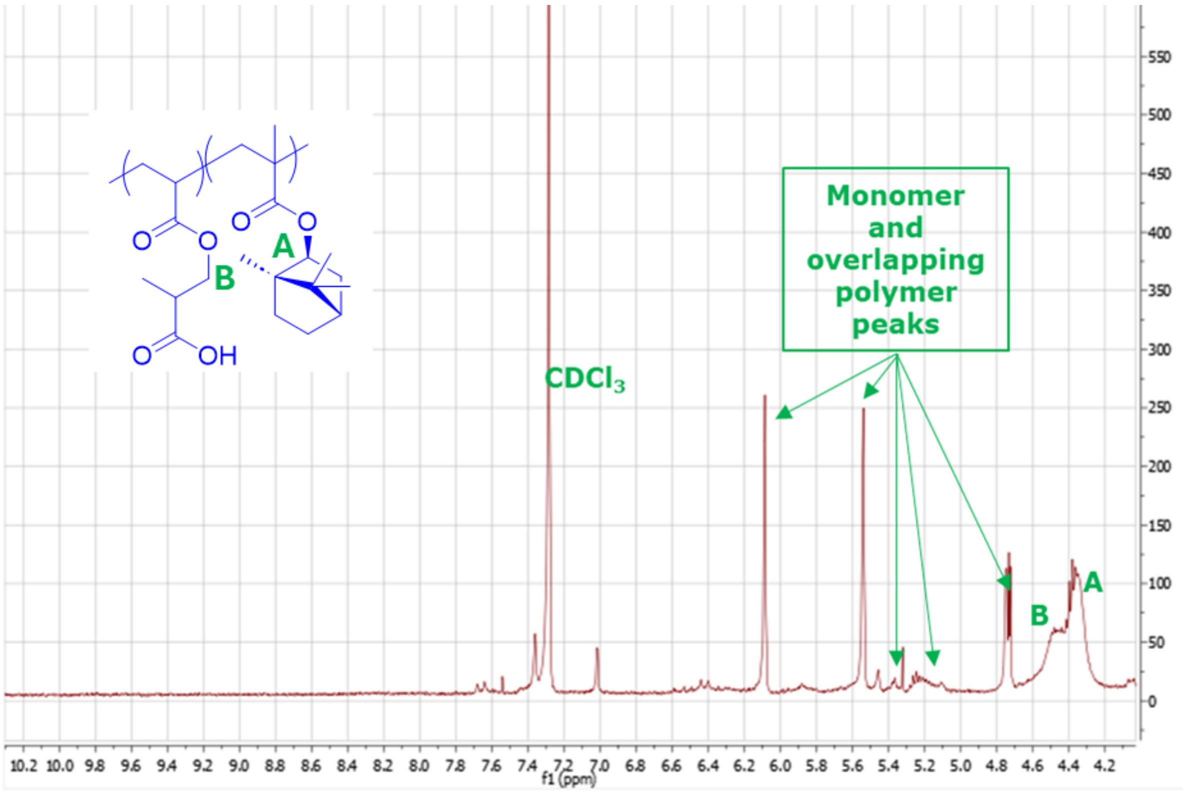

Supplementary Fig 45 - ^{1}H NMR of p(IBMA- modified lactic acid acrylate) outer shell synthesized in 60 mL autoclave ^{1}H NMR (400 MHz, $CHCl_3$-d_3, δ): 4.23-4.60 (mp, 1H, CH), 4.23-4.60 (mp, 2H, CH_2)

Sample	co-polymer formation[a,b]	Mn (KDa)[a]	Mw (KDa)[a]	Đ [a]	T_g[c]	T_m[c]	T_c[c]
PA-12 coated with p(IBMA-bornyl methacrylate) 10wt%	Yes	22	54	2.4	138.37	172.77	140.37
PA-12 coated with p(IBMA-bornyl methacrylate) 20wt%	Yes	22	132	5.879	139.41	174.06	141.92
PA-12 coated with p(IBMA-bornyl methacrylate) 30wt%	Yes	25	56	2.273	139.21	172.77	140.37
PA-12 coated with p(IBMA-cinnamyl methacrylate) 10wt%	Yes	26	114	4.312	140.39	175.36	143.22
PA-12 coated with p(IBMA-cinnamyl methacrylate) 20wt%	Yes	10	46	4.5	139.62	175.62	143.22
PA-12 coated with p(IBMA-cinnamyl methacrylate) 30wt%	Yes	8	44	5.6	142.04	175.62	144.25
PA-12 coated with p(IBMA-neryl methacrylate) 10wt%	Yes	26	70	2.7	142.97	173.29	145.03
PA-12 coated with p(IBMA-neryl methacrylate) 20wt%	Yes	26	86	3.3	144.76	174.32	146.59
PA-12 coated with p(IBMA-neryl methacrylate) 30wt%	Yes	19	47	2.5	147.28	175.36	147.62
PA-12 coated with p(IBMA-myrtenol methacrylate) 10wt%	Yes	26	72	2.9	143.77	171.47	143.99

Sample	co-polymer formation[a,b]	Mn (KDa)[a]	Mw (KDa) [a]	Đ [a]	T_g[c]	T_m[c]	T_c[c]
PA-12 coated with p(IBMA-myrtenol methacrylate) 20wt%	Yes	21	106	5	142.51	171.99	143.22
PA-12 coated with p(IBMA-myrtenol methacrylate) 30wt%	Yes	21	112	5.3	142.92	171.99	143.22
PA-12 coated with p(IBMA-modified oleic acid acrylate) 10wt%	Yes	26	115	4.515	148.28	176.14	148.66
PA-12 coated with p(IBMA-modified oleic acid acrylate) 20wt%	Yes	18	76	4.309	145.62	175.88	148.4
PA-12 coated with p(IBMA-modified oleic acid acrylate) 30wt%	Yes	29	149	5.197	147.97	176.14	149.44
PA-12 coated with p(IBMA-modified lactic acid acrylate) 10wt%	Yes	16	50	3.202	148.52	176.4	149.49
PA-12 coated with p(IBMA-modified lactic acid acrylate) 20wt%	Yes	19	48	2.566	150.36	176.14	151.25
PA-12 coated with p(IBMA-modified lactic acid acrylate) 30wt%	Yes	18	69	3.924	151.22	176.4	151.77

Supplementary Table 7 – Table of data describing the coating of PA-12 with p(IBMA-terpene monomer) and p(IBMA-modified acid acrylate). [a] Determined through GPC analysis of the outer shell extracted from the coated PA-12 particles. [b] Determined through ^{1}H NMR analysis of the outer shell extracted from the coated PA-12 particles. [c] Determined through DSC analysis of coated PA-12 particles.

The printed parts that were bioassayed against the four microorganisms were also tested for toxicity against those microorganisms. These tests were performed to determine if the growth preventative effect might be attributable to leaching of a cytotoxic component (monomer) from the surface, which might inhibit the organism, rather than the prevention of attachment and/or formation of biofilm. (Supplementary Fig 46). From these results it can be concluded that the polymeric coating materials are largely non-toxic to most of the tested organisms, hence the observed effects can be attributed to biofilm-specific development, e.g., cell/spore adhesion processes.

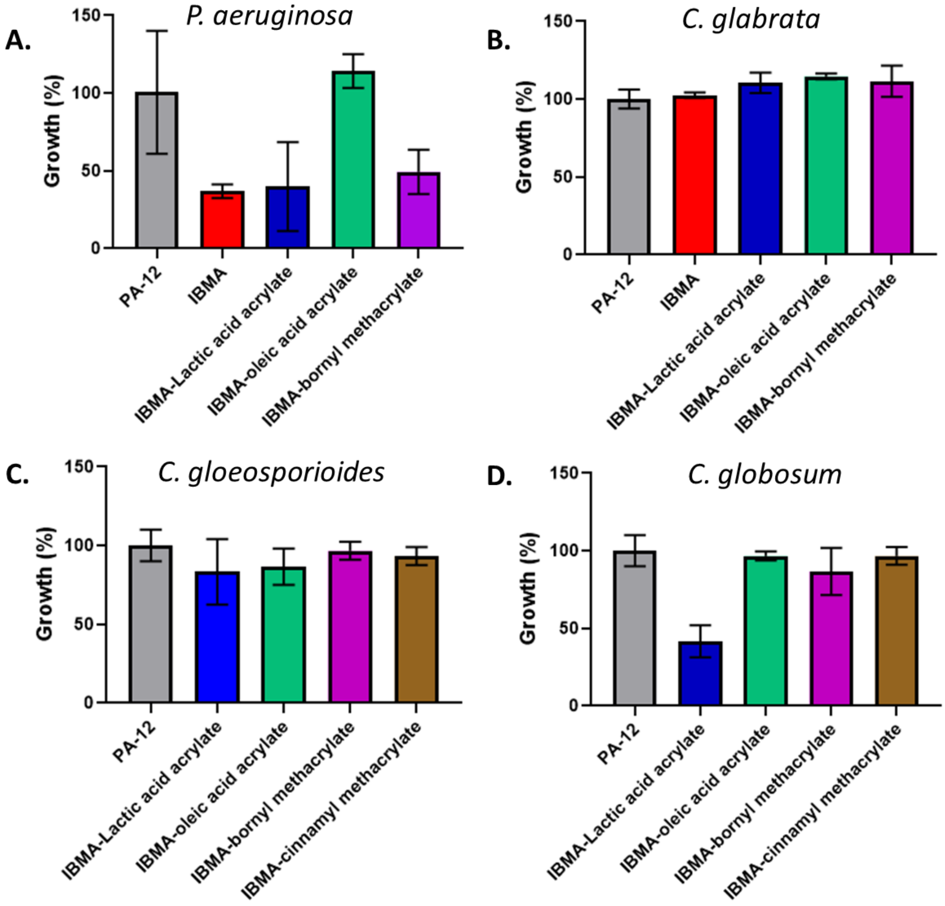

Supplementary Fig 46 – Toxicity tests of the printed discs (1.5 mm in height and 5 mm in diameter) A.) *P. aeruginosa*. B.) *C. glabrata*. C.) *C. gloeosporioides*. D.) *C. globosum.* The data show that for *P. aeruginosa* (S47A) there is a ~60% reduction (in growth in the presence PIBMA, P(IBMA-modified lactic acid acrylate), and ~50% in the case of P(IBMA-bornyl methacrylate), indicating some slowing of growth in these cases. For *C. glabrata*, *C. gloeosporioides* and *C. globosum* the printed polymers are not cytotoxic, with only P(IBMA-modified lactic acid acrylate showing any growth inhibition of *C. globosum*. The absence of any toxicity arising from the majority of the materials is in stark contrast to the clear and evident inhibition of biofilm development (see Figure 4B,C). Values shown are means from at least three biological replicates, with error bars showing standard error of the mean.

Supplementary Information for PBF printing accuracy and variety:

A variety of further parts were printed with both the PA-12 coated with P(IBMA-DB3MA) (blue) and P(IBMA-DR1MA) (red) in varying ratios and on a range of scales and geometries. A design for testing resolution of printed parts was built from a 80/20 mix of virgin PA-12 and PA-12 coated with P(IBMA-DB3MA)(Supplementary Fig 47).

A.

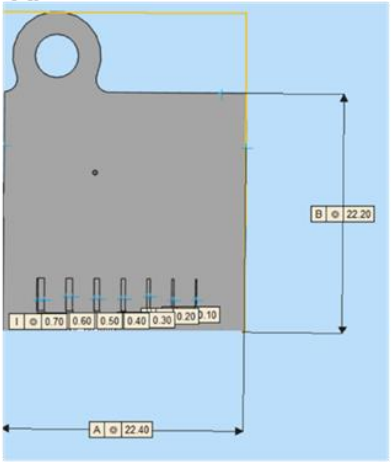

B.

D.

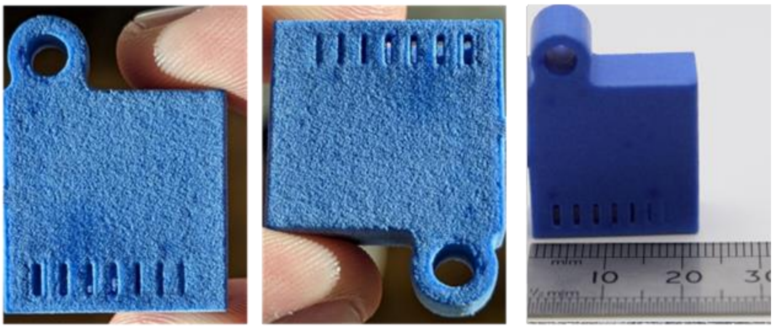

C.

	X	Y	Y2	Z
Part	22.11	22.38	29.48	10.01
Target	22.20	22.40	29.586	10.00

E.

F.

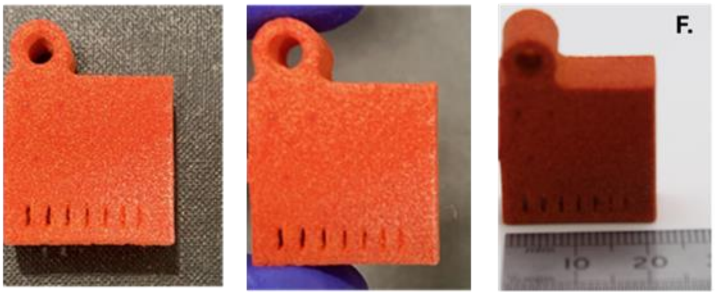

G.

	X	X2	Y	Y2	Z
Part	22.04	27.91	22.26	29.47	9.85
Target	22.20	28.00	22.40	29.586	10.00

Supplementary Fig 47 – A.) CAD model of an industry standard printed resolution test model B.) Printed model built from a 80/20 mix of virgin PA-12 and PA-12 coated with P(IBMA-DB3MA) respectively. C.) Table of actual measurements of the printed part vs the design targets (an 80/20 mix of virgin PA-12 and PA-12 coated with P(IBMA-DB3MA) respectively). D.) Printed model after vapor smoothing composed of an 80/20 mix of virgin PA-12 and PA-12 coated with P(IBMA-DB3MA) respectively using a ruler as the scale bar. The printed parts show a resolution of ~100 μm and an overall error in dimension of less than 0.5%. E.) Printed model built from a PA-12 coated with P(IBMA-DR1MA). F.) Printed model composed of PA-12 coated with P(IBMA-DR1MA) using a ruler as the scale bar. G.) Table of actual measurements of the printed part vs the design targets (PA-12 coated with P(IBMA-DR1MA).

A model race car which has interlocking parts (wheels interlock with the main body of the car) was constructed from a 80/20 mix of virgin PA-12 and PA-12 coated with P(IBMA-DB3MA) respectively (Supplementary Fig 48).

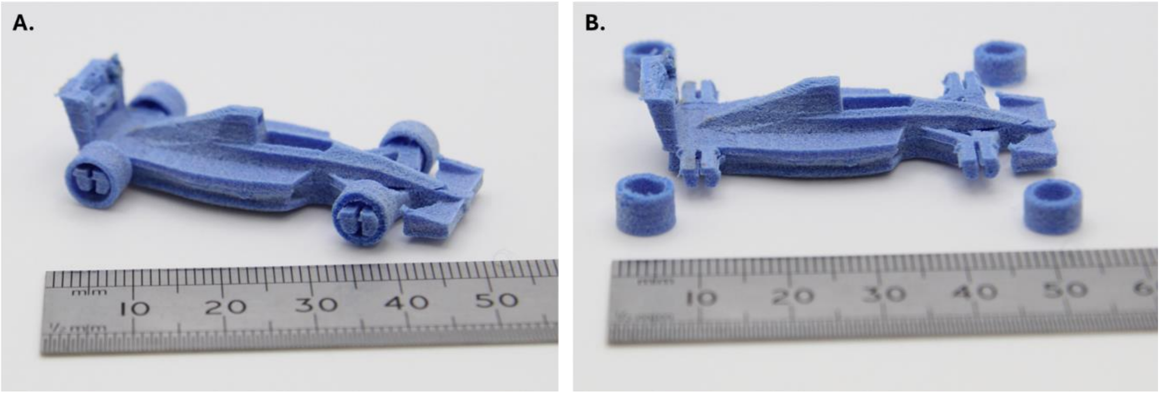

Supplementary Fig 48 – Printed race car model built from a 80/20 mix of virgin PA-12 and PA-12 coated with P(IBMA-DB3MA) respectively. A.) fully assembled. B.) with detachable wheels removed.

Tensile bars were printed from PA-12 coated with P(IBMA-DR1MA) (red) and a series of blends (Supplementary Fig 49).

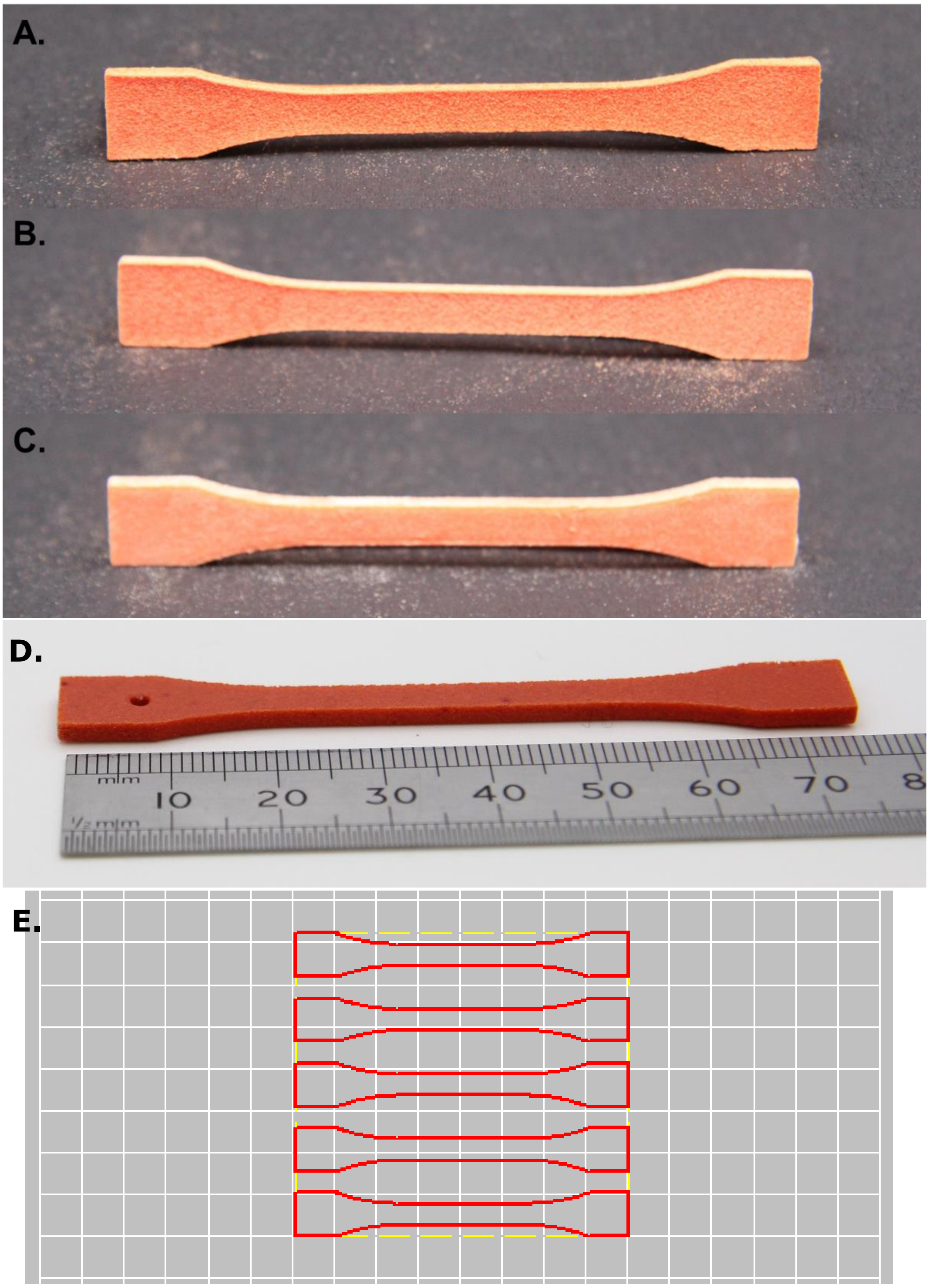

Supplementary Fig 49 – Tensile bars A.) built from PA-12 coated with P(IBMA-DR1MA). B.) from a 50/50 mix of PA-12 coated with P(IBMA-DR1MA) and virgin PA-12. C.) from an 80/20 mix of virgin PA-12 and PA-12 coated with P(IBMA-DR1MA) respectively. D.) Image of a tensile bar built from a 50/50 mix of PA-12 coated with P(IBMA-DR1MA) and virgin PA-12 after smoothing (ruler used as a scale bar). E.) CAD model of the tensile bars showing build orientation.

A lattice structure was built from PA-12 coated with P(IBMA-DR1MA), showing that a complex intertwined part can be built with the coated PA-12 based material (Supplementary Fig 50).

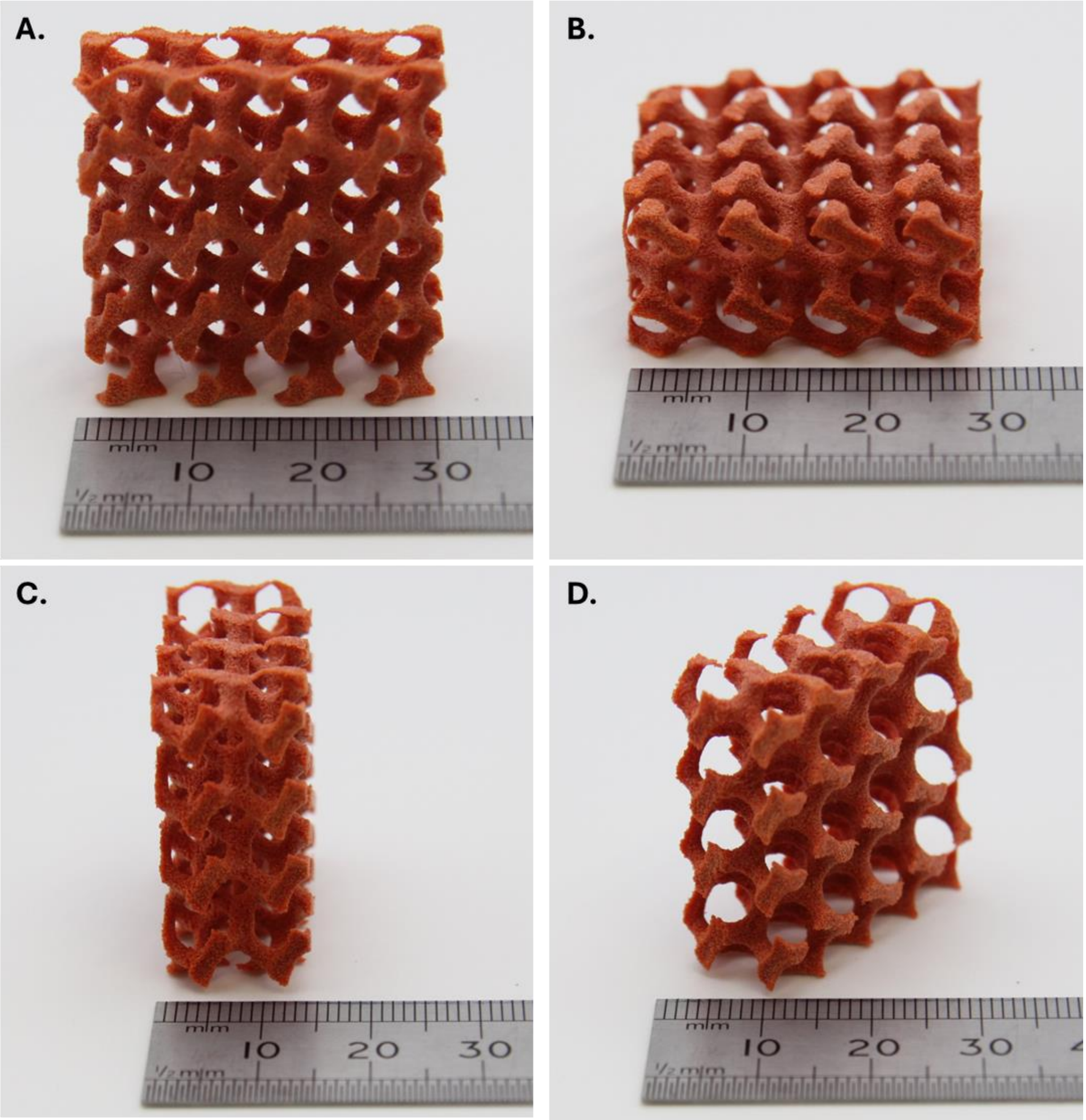

Supplementary Fig 50 – Images of lattice type structure built from PA-12 coated with P(IBMA-DR1MA). A.) Front end view. B.) Top view C.) Side end view D.) Diagonal view.

An industry standard surface finish can be achieved following vapour smoothing (commonly used post-processing tool). Vapour smoothing was used on a toy block printed with an 80/20 mix of virgin PA-12 and PA-12 coated with P(IBMA-DB3MA) respectively (Supplementary Fig 51).

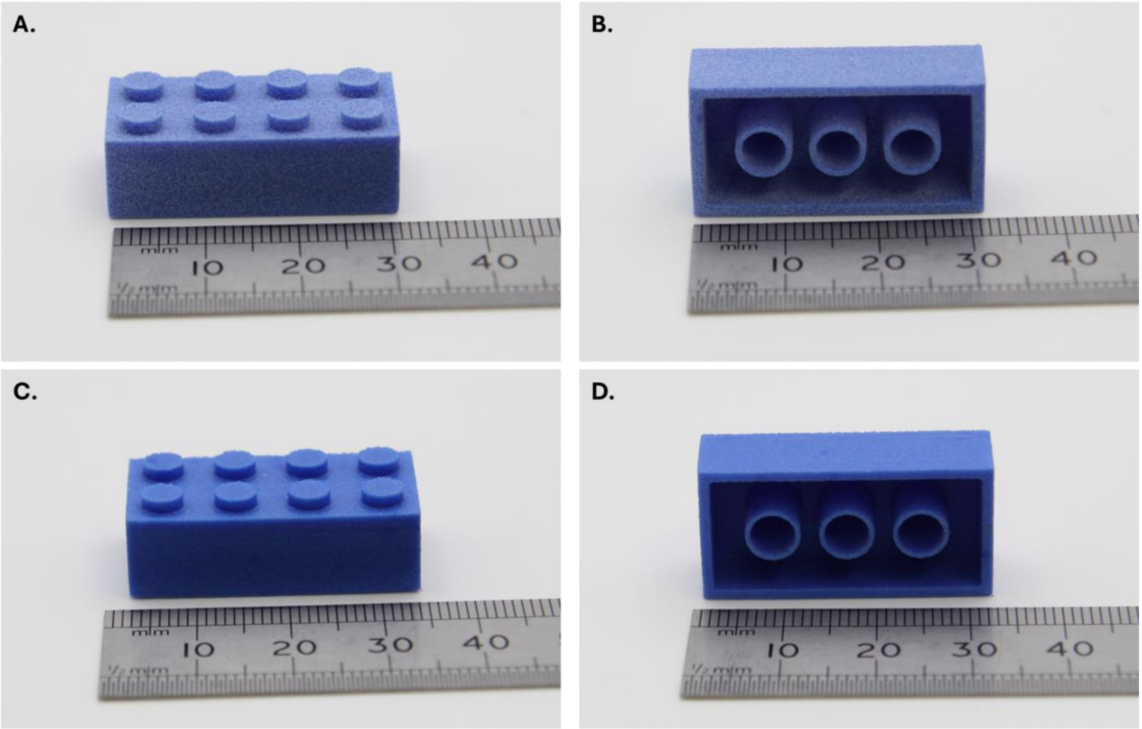

Supplementary Fig 51 – Images of non-smoothed and post vapour smoothed printed parts composed of an 80/20 mix of virgin PA-12 and PA-12 coated with P(IBMA-DB3MA) showing an improvement in surface finish. A.) Top view of non-smoothed part. B.) Bottom view of non-smoothed part. C.) Top view of smoothed part. D.) Bottom view of smoothed part.

Supporting Information for the mechanical properties of the printed parts:

Mechanical testing was conducted on tensile bars printed using ISO-527-2 standards. These standards are known to be equivalent to ASTM D638 standards. Both are the international standard for tensile testing of rigid and semi rigid thermoplastic molded, extruded, and cast materials. Four differing compositions were chosen:

- 100% virgin PA-12 (control)
- 100% PA-12 coated with P(IBMA-DR1MA) (Red)
- 50/50 % mix of two powders; PA-12 coated with P(IBMA-DR1MA) and virgin PA-12
- 80/20 % mix of two powders; virgin PA-12 and PA-12 coated with P(IBMA-DR1MA) respectively

The only difference in printing conditions of the tensile bars was heating rate, as the powders containing PA-12 coated with P(IBMA-DR1MA) necessitated a slower heating rate (1.5 min/degree compared to 1 min/degree) to prevent curling. Having the same sintering conditions allows for reliable mechanical property comparisons of the printed objects. All of the tensile bars were printed flat with the long axis in X orientation (Supplementary Fig 49D). Testing revealed that mechanical properties are altered through the addition of the functional coatings (Supplementary Fig 52).

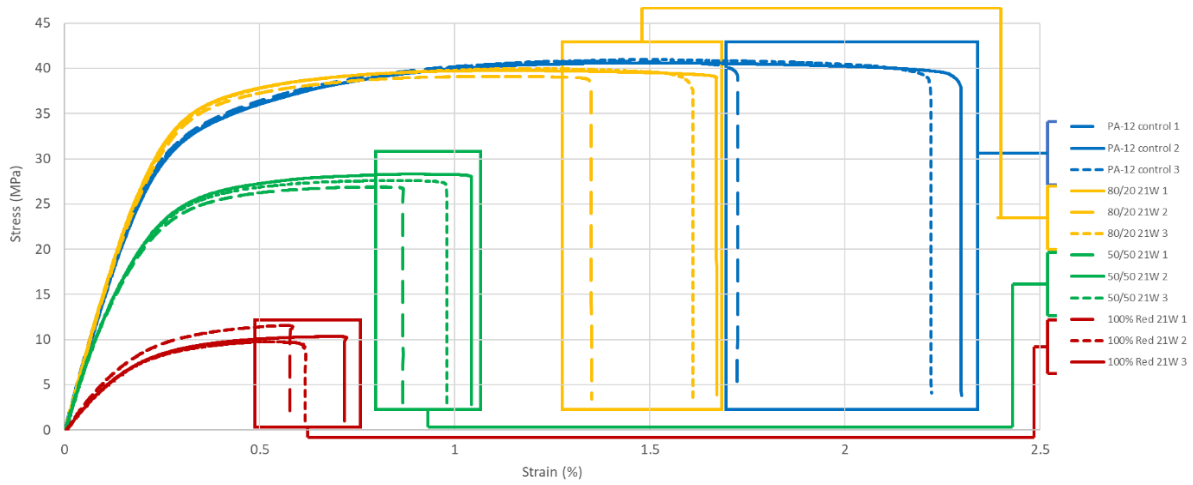

Supplementary Fig 52 – Mechanical properties testing (Stress vs Strain) using ISO 527-2 standard tensile bars composed of four differing compositions, 100% virgin PA-12 (control; blue group) and three coloured samples built from 100% PA-12 coated with P(IBMA-DR1MA)(dark red group), a 50/50 % mix of PA-12 coated with P(IBMA-DR1MA) and virgin PA-12 (green group), and an 80/20 % mix of virgin PA-12 and PA-12 coated with P(IBMA-DR1MA) respectively (yellow group).

	PA-12 1	PA-12 2	PA-12 3	80/20 mix 1	80/20 mix 2	80/20 mix 3
Tensile stress at maximum load (MPa)	40.76	40.6	40.96	39.77	39.11	39.95
Tensile strain at break (%)	2.29	1.72	2.22	1.67	1.35	1.61

Supplementary Table 8 – Summary of individual mechanical testing results (in triplicate) for parts printed with commercial PA-12 and parts printed with an 80/20 mix of

commercial PA-12 and PA-12 coated with P(IBMA-DR1MA). Parts tested with ISO-527-2 protocols.

Supplementary Information for surface roughness analysis of printed parts:

A variety of printed samples were analysed:

- Tensile bar composed of PA-12 coated with P(IBMA-DR1MA)(Red)
- Tensile bar composed of a 50/50 mix of PA-12/PA-12 coated with P(IBMA-DR1MA)
- Tensile bar composed of an 80/20 mix of PA-12/PA-12 coated with P(IBMA-DR1MA)
- Tensile bar composed of PA-12
- Vapour Smoothed Tensile bar composed of an 80/20 mix of PA-12/PA-12 coated with P(IBMA-DR1MA)
- Vapour Smoothed Tensile bar composed of PA-12

The area measured was a single field of view (2858 mm x 2176 mm) of two different locations in every specimen, one at the tab of the sample and another at the centre. Alicona G5 surface texture measurements were performed (mode: 5X objective, vertical resolution of 1.50 mm, lateral resolution of 14.70 mm, z-vertical focus variation from -700 mm to +700 mm.).

Samples	Sq (μm)	Ssk	Sku	Sp (μm)	Sv (μm)	Sz (μm)	Sa (μm)
Red non-smoothed (tab)	29.75	-0.7814	4.78	96.3	193.1	289.4	22.79
Red non-smoothed (center)	36.68	-0.6145	3.882	132.9	202.5	338.4	28.95
50/50 non-smoothed (tab)	9.263	0.2374	4.35	68.7	39.81	108.5	7.137
50/50 non-smoothed (center)	10.97	0.08256	4.754	101.4	62	163.4	8.459
80/20 not-smoothed (tab)	11.69	0.5064	3.66	78.66	41.56	120.2	9.207
80/20 not-smoothed (center)	11.55	0.244	3.449	55.71	50.7	106.4	9.07
PA-12 non-smoothed (tab)	13.93	0.2435	2.879	57.66	45.4	103	11.19
PA-12 non-smoothed (center)	17.51	0.4617	3.09	70.8	45.38	116.2	13.96
80/20 smoothed (tab)	2.332	-0.07847	3.643	16.12	10.6	26.72	1.825
80/20 smoothed (center)	2.206	-0.1902	4.479	13.06	13.5	26.5	1.696
PA-12 smoothed (tab)	1.815	-0.4627	4.338	10.25	12.07	22.32	1.401
PA-12 smoothed (center)	1.878	-0.6198	4.856	9.46	10.98	20.44	1.418

Supplementary Table 9 – Results of surface roughness analysis of a variety of printed parts. Sq: root mean square height. Ssk: skewness. Sku: Kurtosis. Sp: maximum peak height. Sv: maximum pit height. Sz: Maximum height (sum of maximum peak height and maximum pit height). Sa: arithmetical mean height.

The results show that the 'non-smoothed' 80/20 mix printed parts without post processing yielded components that were smoother than their control counterparts from virgin PA-12 (Supplementary Tables 10 and 11), as the Sq and Sa values for the 'non-smoothed' 80/20 mix printed parts are lower than the control and the Sp and Sv are in

the same range. Again, this suggests that the 80/20 mix material is highly suited to laser sintering processes.

	Mean of 80/20 mix 'non-smoothed' tab	Mean of 80/20 mix 'non-smoothed' centre	Mean of total 80/20 mix 'non-smoothed '	Range
Sq (µm)	11.68	11.54	11.61	11.54-11.69
Sp (µm)	78.68	56.21	67.45	55.71-78.73
Sv (µm)	41.4	50.73	46.07	41.28-50.99
Sa (µm)	9.19	9.062	9.13	9.06-9.21

Supplementary Table 10 – Topology and surface roughness of "non-smoothed" printed samples composed of an 80/20 mix of commercial virgin PA-12 and PA-12 coated with P(IBMA-DR1MA).

	Mean of PA-12 'non-smoothed' tab	Mean of PA-12 'non-smoothed' centre	Mean of total PA-12 'non-smoothed '	Range
Sq (µm)	13.93	17.5	15.72	13.91-17.51
Sp (µm)	58.49	70.61	64.55	57.60-70.80
Sv (µm)	45.15	45.51	45.33	44.96-45.85
Sa (µm)	11.19	13.95	12.57	11.17-13.96

Supplementary Table 11 – Topology and surface roughness of "non-smoothed" printed samples composed of control PA-12.

Carrying out the same analysis for the 'smoothed' 80/20 mix printed samples. These are slightly less smooth compared to the control PA-12 samples as Sq, Sp, Sv, Sa values are higher than those of the control (Supplementary Tables 12 and 13). The increase in surface roughness between the 80/20 mix and the commercial virgin PA-12 after smoothing could be because the same "smoothing" procedure was utilized for both materials. This 'smoothing' procedure has been industrially optimized for commercial PA-12; therefore, it is probable that with "smoothing" procedure optimization for the 80/20 mix the differences would become smaller.

	Mean of 80/20 mix 'smoothed' tab	Mean of 80/20 mix 'smoothed' centre	Mean of total 80/20 mix 'smoothed '	Range
Sq (µm)	2.33	2.2	2.27	2.20-2.33
Sp (µm)	16.23	13.32	14.78	13.00-16.38
Sv (µm)	10.67	13.45	12.06	10.60-13.50
Sa (µm)	1.83	1.7	1.77	1.67-1.83

Supplementary Table 12 – Topology and surface roughness of "smoothed" printed samples composed of an 80/20 mix of commercial virgin PA-12 and PA-12 coated with P(IBMA-DR1MA).

	Mean of PA-12 'smoothed' tab	Mean of PA-12 'smoothed' centre	Mean of total PA-12 'smoothed '	Range
Sq (µm)	1.82	1.88	1.85	1.81-1.88
Sp (µm)	9.93	9.48	9.71	9.46-10.25
Sv (µm)	12.26	11.04	11.65	10.98-12.41
Sa (µm)	1.4	1.42	1.41	1.40-1.42

Supplementary Table 13 – Topology and surface roughness of "smoothed" printed samples composed of a control PA-12.

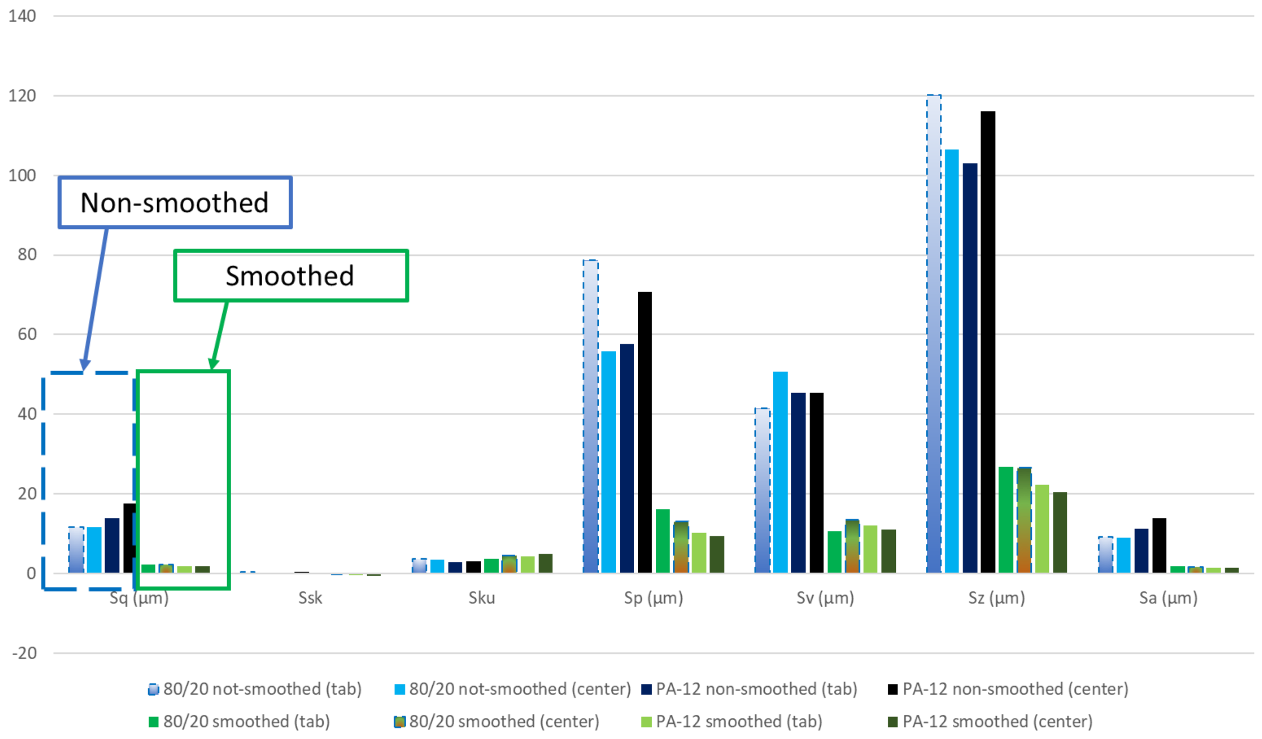

Supplementary Fig 53 – Comparison of surface roughness measurements of printed parts composed of a variety of materials.

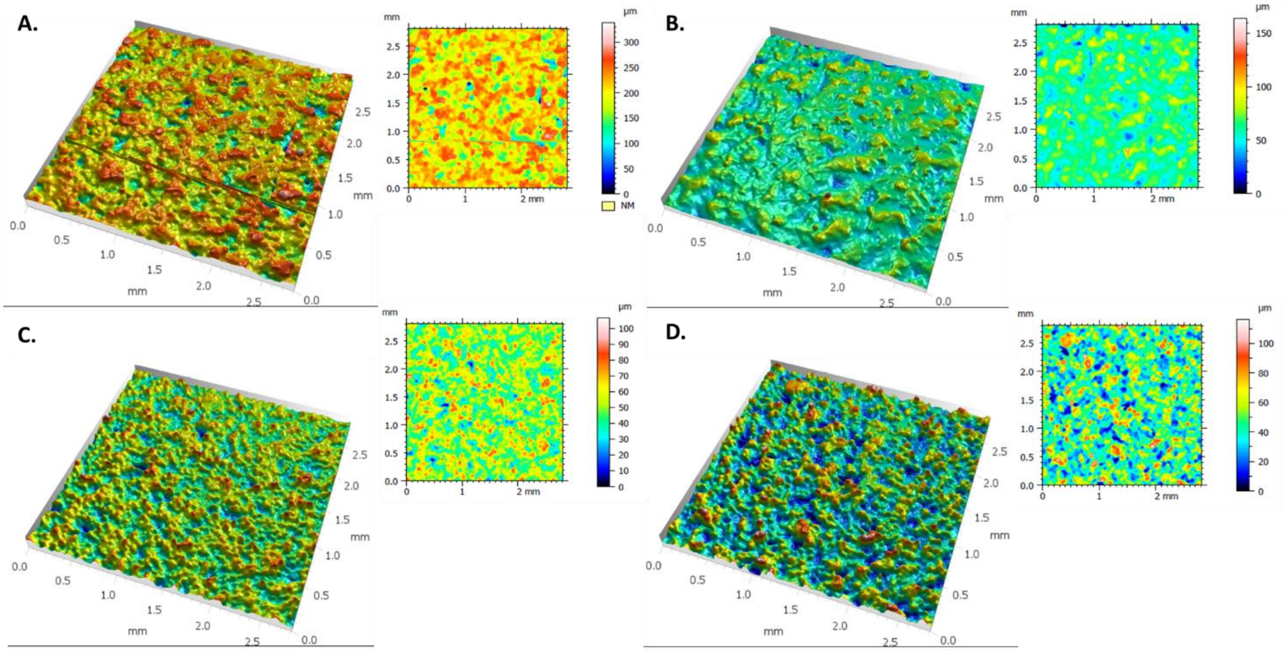

Supplementary Fig 54 – Surface roughness results and mapping of printed parts. A.) Measurement and surface mapping for part composed of Red PA-12 (PA-12 coated with P(IBMA-DR1MA)). B.) Measurement and surface mapping for part composed of a 50/50 mix of 50% White PA-12 and 50% Red PA-12 (PA-12 coated with P(IBMA-DR1MA)). C.) Measurement and surface mapping for part composed of a 80/20 mix of 80% White PA-12 and 20% Red PA-12 (PA-12 coated with P(IBMA-DR1MA)). D.) Measurement and surface mapping for part composed of PA-12.

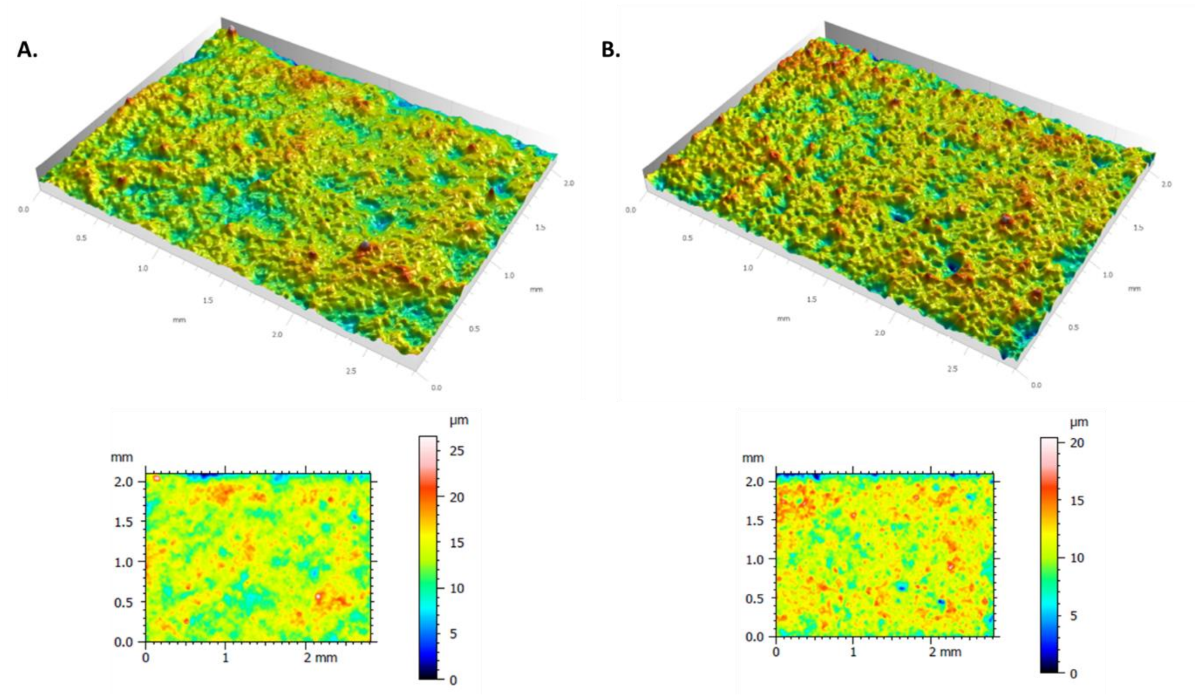

Supplementary Fig 55 – Surface roughness results and mapping of smoothed printed parts. A.) Measurement and surface mapping for smoothed part composed of a 80/20 mix of 80% White PA-12 and 20% Red PA-12 (PA-12 coated with P(IBMA-DR1MA)). B.) Measurement and surface mapping for smoothed part composed of PA-12.

Supporting Information for the hydrophobicity of printed parts:

Printed parts constructed from three materials and assessed via water contact angle measurements:

- Virgin PA-12,
- PA-12 particles coated with P(IBMA-DR1MA)(Red),
- PA-12 particles coated with P(IBMA-DB3MA)(Blue).

These data reveal that there was a difference in hydrophobicity based upon the dye monomer and its structure (Supplementary Table 14). In the sample composed of PA-12 coated with P(IBMA-DR1MA) the dye monomer is at 10 wt% (with respect to IBMA) and DR1MA is more hydrophobic, therefore the water contact angle is bigger than for the control of PA-12. Conversely the sample coated with P(IBMA-DB3MA) appears more hydrophilic, but the effect is smaller because DB3MA was used at a loading of only 2.5 wt% (with respect to IBMA) in that coating.

**Sample**	**Water Contact Angle (°)**
PA-12	85.4
Red PA-12 coated with P(IBMA-DR1MA)	107.1
Blue PA-12 coated with P(IBMA-DB3MA)	81.1

Supplementary Table 14 **–** Water contact angle for the surface of printed parts.

Supplementary References

1. Haddleton, A., Bassett, S. & Howdle, S. Comparison of Polymeric Particles Synthesised Using scCO2 as the Reaction Medium on the Millilitre and Litre Scale. *J. Supercrit. Fluids* **160**, 104785 (2020).
2. Luo, M. R., Cui, G. & Rigg, B. The development of the CIE 2000 colour-difference formula: CIEDE2000. *Color Res. Appl.* **26**, 340–350 (2001).
3. Kennedy, J. & Eberhart, R. Particle swarm optimization. in *Proceedings of ICNN'95 - International Conference on Neural Networks* **4**, 1942–1948 vol.4 (1995).
4. Shi, Y. & Eberhart, R. A modified particle swarm optimizer. in *1998 IEEE International Conference on Evolutionary Computation Proceedings. IEEE World Congress on Computational Intelligence (Cat. No.98TH8360)* 69–73 (1998). doi:10.1109/ICEC.1998.699146
5. Cornell, J. *A Primer on Experiments with Mixtures*. (2011). doi:10.1002/9780470907443
