# Supplementary material for: A facile one step route that introduces functionality to polymer powders for laser sintering
Source: Nat Commun. 2024 Apr 11;15:3137. doi: 10.1038/s41467-024-47376-4 (PMC11009337; doi:10.1038/s41467-024-47376-4)
Supplement: Supplementary file 3 — Reporting Summary [file 41467_2024_47376_MOESM3_ESM.pdf]

nature portfolio

Corresponding author(s): Steven M. Howdle

Last updated by author(s): 19/03/24

Reporting Summary

Nature Portfolio wishes to improve the reproducibility of the work that we publish. This form provides structure for consistency and transparency in reporting. For further information on Nature Portfolio policies, see our Editorial Policies and the Editorial Policy Checklist.

Statistics

For all statistical analyses, confirm that the following items are present in the figure legend, table legend, main text, or Methods section.

n/a	Confirmed	
☐	☒	The exact sample size (*n*) for each experimental group/condition, given as a discrete number and unit of measurement
☐	☒	A statement on whether measurements were taken from distinct samples or whether the same sample was measured repeatedly
☐	☒	The statistical test(s) used AND whether they are one- or two-sided<br>*Only common tests should be described solely by name; describe more complex techniques in the Methods section.*
☐	☒	A description of all covariates tested
☐	☒	A description of any assumptions or corrections, such as tests of normality and adjustment for multiple comparisons
☐	☒	A full description of the statistical parameters including central tendency (e.g. means) or other basic estimates (e.g. regression coefficient) AND variation (e.g. standard deviation) or associated estimates of uncertainty (e.g. confidence intervals)
☐	☒	For null hypothesis testing, the test statistic (e.g. *F*, *t*, *r*) with confidence intervals, effect sizes, degrees of freedom and *P* value noted<br>*Give P values as exact values whenever suitable.*
☒	☐	For Bayesian analysis, information on the choice of priors and Markov chain Monte Carlo settings
☒	☐	For hierarchical and complex designs, identification of the appropriate level for tests and full reporting of outcomes
☒	☐	Estimates of effect sizes (e.g. Cohen's *d*, Pearson's *r*), indicating how they were calculated

Our web collection on statistics for biologists contains articles on many of the points above.

Software and code

Policy information about availability of computer code

Data collection

For the prediction of the colour of printed parts a combination of Particle Swarm Optimisation (PSO)1-3 and simplex-lattice/centroid designs was used. The prediction model (multivariate cubic regression model) was built based on a 4-factor simplex-lattice design generated by using the TIBCO Statistica® 13.3.0. software. The Statistical evaluation with a significance level (p) equal to 0.05 and the parametric data analysis based on the analysis of variance (ANOVA) were also carried out using the TIBCO Statistica® 13.3.0.

The sequential optimisation procedure adopted to determine the colour composition based on PSO technique finds the best candidates for the cubic regression model by minimization of quadratic difference between the desired value of the colour difference and response (output) of the cubic regression model used to evaluate the optimal estimated composition values for the components (colours). The computer codes for the optimisation tasks were written in both Fortran (Intel® Fortran Compiler 19.0 for Windows) and Python (Python 3.10.6 64-bit for macOS) and are available on request and/or from GitHub (https://github.com/faumachado/colour_model).

Data analysis

For the prediction of the colour of printed parts a combination of Particle Swarm Optimisation (PSO)1-3 and simplex-lattice/centroid designs was used. The prediction model (multivariate cubic regression model) was built based on a 4-factor simplex-lattice design generated by using the TIBCO Statistica® 13.3.0. software. The Statistical evaluation with a significance level (p) equal to 0.05 and the parametric data analysis based on the analysis of variance (ANOVA) were also carried out using the TIBCO Statistica® 13.3.0.

The sequential optimisation procedure adopted to determine the colour composition based on PSO technique finds the best candidates for the cubic regression model by minimization of quadratic difference between the desired value of the colour difference and response (output) of the cubic regression model used to evaluate the optimal estimated composition values for the components (colours). The computer codes for the optimisation tasks were written in both Fortran (Intel® Fortran Compiler 19.0 for Windows) and Python (Python 3.10.6 64-bit for

macOS) and are available on request and/or from GitHub (https://github.com/faumachado/colour_model).

For manuscripts utilizing custom algorithms or software that are central to the research but not yet described in published literature, software must be made available to editors and reviewers. We strongly encourage code deposition in a community repository (e.g. GitHub). See the Nature Portfolio guidelines for submitting code & software for further information.

Data

Policy information about availability of data

All manuscripts must include a data availability statement. This statement should provide the following information, where applicable:

- Accession codes, unique identifiers, or web links for publicly available datasets
- A description of any restrictions on data availability
- For clinical datasets or third party data, please ensure that the statement adheres to our policy

Supporting information: includes materials, synthetic and analysis methods, and further characterisation. The full data is available from the corresponding author upon request.

Research involving human participants, their data, or biological material

Policy information about studies with human participants or human data. See also policy information about sex, gender (identity/presentation), and sexual orientation and race, ethnicity and racism.

Reporting on sex and gender	NA
Reporting on race, ethnicity, or other socially relevant groupings	NA
Population characteristics	NA
Recruitment	NA
Ethics oversight	NA

Note that full information on the approval of the study protocol must also be provided in the manuscript.

Field-specific reporting

Please select the one below that is the best fit for your research. If you are not sure, read the appropriate sections before making your selection.

☒ Life sciences ☐ Behavioural & social sciences ☐ Ecological, evolutionary & environmental sciences

For a reference copy of the document with all sections, see nature.com/documents/nr-reporting-summary-flat.pdf

Life sciences study design

All studies must disclose on these points even when the disclosure is negative.

Sample size	At least three independent biological replicates were used. This followed our previous work (e.g. https://www.science.org/doi/full/10.1126/sciadv.aba6574) establishing this to be an appropriate sample size for determining statistically significant effects for the assays described.
Data exclusions	No data were excluded
Replication	The use of independent biological replicates (see above) was used to assure experimental reproducibility. All experiments were replicated and all attempts at replication are presented. At least three independent biological replicates were used. This followed our previous work (e.g. https://www.science.org/doi/full/10.1126/sciadv.aba6574) establishing this to be an appropriate sample size for determining statistically significant effects for the assays described.
Randomization	Data were collected by test-material, for each test-organism. All biological measurements were continuous rather than discrete. The experiments were grouped by the organisms, which were independent of each other and therefore covariates were irrelevant.
Blinding	Blinding was not used as the researchers needed to determine which samples were the control groups for data analysis and additionally, the control group PA-12 was visibly different to the other samples.

Reporting for specific materials, systems and methods

We require information from authors about some types of materials, experimental systems and methods used in many studies. Here, indicate whether each material, system or method listed is relevant to your study. If you are not sure if a list item applies to your research, read the appropriate section before selecting a response.

Materials & experimental systems

n/a	Involved in the study
☒	☐ Antibodies
☒	☐ Eukaryotic cell lines
☒	☐ Palaeontology and archaeology
☐	☒ Animals and other organisms
☒	☐ Clinical data
☒	☐ Dual use research of concern
☒	☐ Plants

Methods

n/a	Involved in the study
☒	☐ ChIP-seq
☒	☐ Flow cytometry
☒	☐ MRI-based neuroimaging

Animals and other research organisms

Policy information about studies involving animals; ARRIVE guidelines recommended for reporting animal research, and Sex and Gender in Research

Laboratory animals	No laboratory animals were used. Only microorganisms.
Wild animals	N/A
Reporting on sex	N/A
Field-collected samples	N/A
Ethics oversight	N/A

Note that full information on the approval of the study protocol must also be provided in the manuscript.
